# Coverable Self‐Cleaning Glass via Abnormal Transport and Jump of Charged Particles

**DOI:** 10.1002/advs.202509404

**Published:** 2025-07-26

**Authors:** Meng Yang, Conglin Li, Wei Tang, Wenchao Gao, Weiguo Weng, Yingchun Wu, Yifan Wang, Chenghang Zheng, Xiang Gao

**Affiliations:** ^1^ State Key Laboratory of Clean Energy Utilization State Environmental Protection Engineering Center for Coal‐Fired Air Pollution Control Zhejiang University Hangzhou 310027 P. R. China; ^2^ State Key Laboratory of Fluid Power and Mechatronic Systems Zhejiang University Hangzhou 310027 P. R. China; ^3^ Beijing Institute of Nanoenergy and Nanosystems Chinese Academy of Sciences Beijing 100083 P. R. China; ^4^ Jiaxing Research Institute Zhejiang University Jiaxing 314031 P. R. China; ^5^ Institute of Carbon Neutrality Zhejiang University Hangzhou 310027 P. R. China; ^6^ Zhejiang Baima Lake Laboratory Co., Ltd. Hangzhou 310051 P. R. China

**Keywords:** force analysis, particle jump, particle transport, photovoltaic power generation, surface self‐cleaning

## Abstract

Surface particle pollution is present everywhere, from windows in daily life to transparent curtain walls in tall buildings and photovoltaic panels in deserts. Cleaning these particles takes a lot of labor and costs, highlighting a method for dealing with surface particle pollution that is facile, fast, and sustainable. Here, the abnormal reverse lateral transport behavior of charged particles is reported, the mechanism of abnormal transport and jump behaviors is revealed of charged particles, and the phase diagram of the influence of electric field and particle size on these two behaviors is established. Based on this discovery, a transparent and simple self‐cleaning glass driven by the electric field is developed, with a thickness of only 0.62 mm, which can achieve a sustainable surface self‐cleaning function when facing organic and inorganic particles. Its cleaning time is ultra‐fast, and 97.79 g m^−2^ particles can be cleaned in 10 s. The self‐cleaning glass can be easily covered to any object surface to achieve self‐cleaning and does not affect the function of the object surface, opening a door for no water consumption, facile, fast, and sustainable self‐cleaning of various surfaces.

## Graphical abstract

The abnormal reverse lateral transport behavior of charged particles is reported. The mechanism of transport and jump behaviors of charged particles is revealed. Furthermore, a transparent and simple self‐cleaning glass driven by the electric field is developed. This study provides the possibility for efficient surface self‐cleaning and opens up new opportunities for directional transport, separation, in situ utilization of particles.

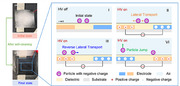

## 1 Introduction

Due to both anthropogenic activities as well as natural processes, surface particle pollution has become a common and prominent problem in many fields such as urban construction,^[^ ^1^ ^]^ energy facilities,^[^ ^2^ ^]^ spacecraft^[^ ^3^ ^]^ and pharmaceutical production.^[^ ^4^ ^]^ A typical example is the problem of dust accumulation on the surface of a photovoltaic panel.^[^ ^5^, ^6^, ^7^ ^]^ It is found that dust accumulation of 5 mg cm^−2^ can cause ≈50% loss of power output of photovoltaic panels.^[^ ^8^ ^]^ In places with high levels of atmospheric pollutants such as India, China, and the Middle East‐North Africa region, solar energy production will be lost by 17–25% per year due to surface particle pollution of photovoltaic panels.^[^ ^9^ ^]^ At present, the cleaning of surface particles still relies on water and simple tools for manual cleaning, resulting in low cleaning efficiency, fresh water consumption, high labor costs, and potential risks to the safety of cleaners.^[^ ^10^, ^11^ ^]^ And there are potential risks to the safety of cleaners, such as the cleaning of tall buildings windows. In addition, the detergent used in the cleaning process may also cause damage to the human body and the ecological environment.^[^ ^12^ ^]^ Therefore, it is urgent to develop facile, fast, and sustainable cleaning methods for surface particles.

Based on the imitation of biological surfaces such as lotus leaves and cicada wings in nature, many researchers have designed and developed nonwetting superhydrophobic surfaces to achieve surface self‐cleaning using droplets.^[^ ^13^, ^14^, ^15^ ^]^ According to the way in which droplets and particles are shed from the surface, the self‐cleaning mechanism of this method can be mainly divided into three types. The first mechanism is that the droplets impact the surface and take away the particles by sliding or rolling.^[^ ^16^, ^17^ ^]^ The second mechanism is that coalescence‐induced jump behaviors of microscale surface condensate droplets take away the particles around them during fogging or condensation.^[^ ^18^, ^19^, ^20^ ^]^ The third mechanism is that a microscale particle (≈10 to≈100 µm) and a droplet can coalesce and self‐transport on superhydrophobic surfaces.^[^ ^21^ ^]^ Although the above self‐cleaning methods have been proved to be effective and ubiquitous in nature, there are still limitations on both droplet size and particle size.^[^ ^13^, ^21^ ^]^ In other words, these methods are very dependent on ambient humidity and surface droplets.^[^ ^22^ ^]^ As a result, the available scenarios of self‐cleaning methods based on nonwetting surfaces are very limited. For example, studies have shown that the superhydrophobic surface in water‐free harsh environments such as deserts is passive and uncontrollable, and its life cannot meet expectations due to fragile surface textures and poor resistance to wear.^[^ ^23^, ^24^ ^]^ Recently, an elastomeric bioinspired stiffness‐gradient catapult inspired by honeybees was designed as a self‐cleaning cleaning robot for solar panels, which shows a ≈91% repellent fraction for 25 µm SiO_2_ particles.^[^ ^25^ ^]^ This method provided a reference and possibility for realizing surface self‐cleaning through solid repulsion.

One of the most promising and feasible methods to achieve surface self‐cleaning is to drive the spontaneous movement of particles through an electric field.^[^ ^26^ ^]^ This method has been successfully applied to various flue gas treatment devices, such as the electrostatic precipitator, which is utilized to reduce particle concentrations in flue gas from coal‐fired power plants.^[^ ^27^ ^]^ Although the particle removal efficiency of an electrostatic precipitator can reach 99%,^[^ ^28^ ^]^ it mainly supports the particle removal in the gas and is powerless for surface particle removal. Electrodynamic screen is an exciting electrostatic cleaning method for surface particle removal. The typical structure of an electrodynamic screen device is to embed a metal wire electrode into plate and activate it with electrical signal to generate standing wave or traveling wave electric field.^[^ ^29^ ^]^ Surface particles can be propelled by the electric field via the dielectrophoretic force or the Coulomb force. Due to the advantages of no water consumption, elimination of touch scrubbing damage, and extremely low energy consumption, the electrodynamic screen has attracted wide attention and has tried to apply on the solar panel of the on Mars rovers.^[^ ^30^ ^]^ In spite of various relevant simulations^[^ ^31^, ^32^, ^33^ ^]^ and experiments,^[^ ^34^, ^35^, ^36^, ^37^ ^]^ the mechanism of how surface particles begin to move and separate from the surface is still unknown, which leads to the lack of structural design criteria for surface self‐cleaning devices^[^ ^38^ ^]^ and the challenges of fine surface particle removal.^[^ ^39^ ^]^

**Figure 1:**
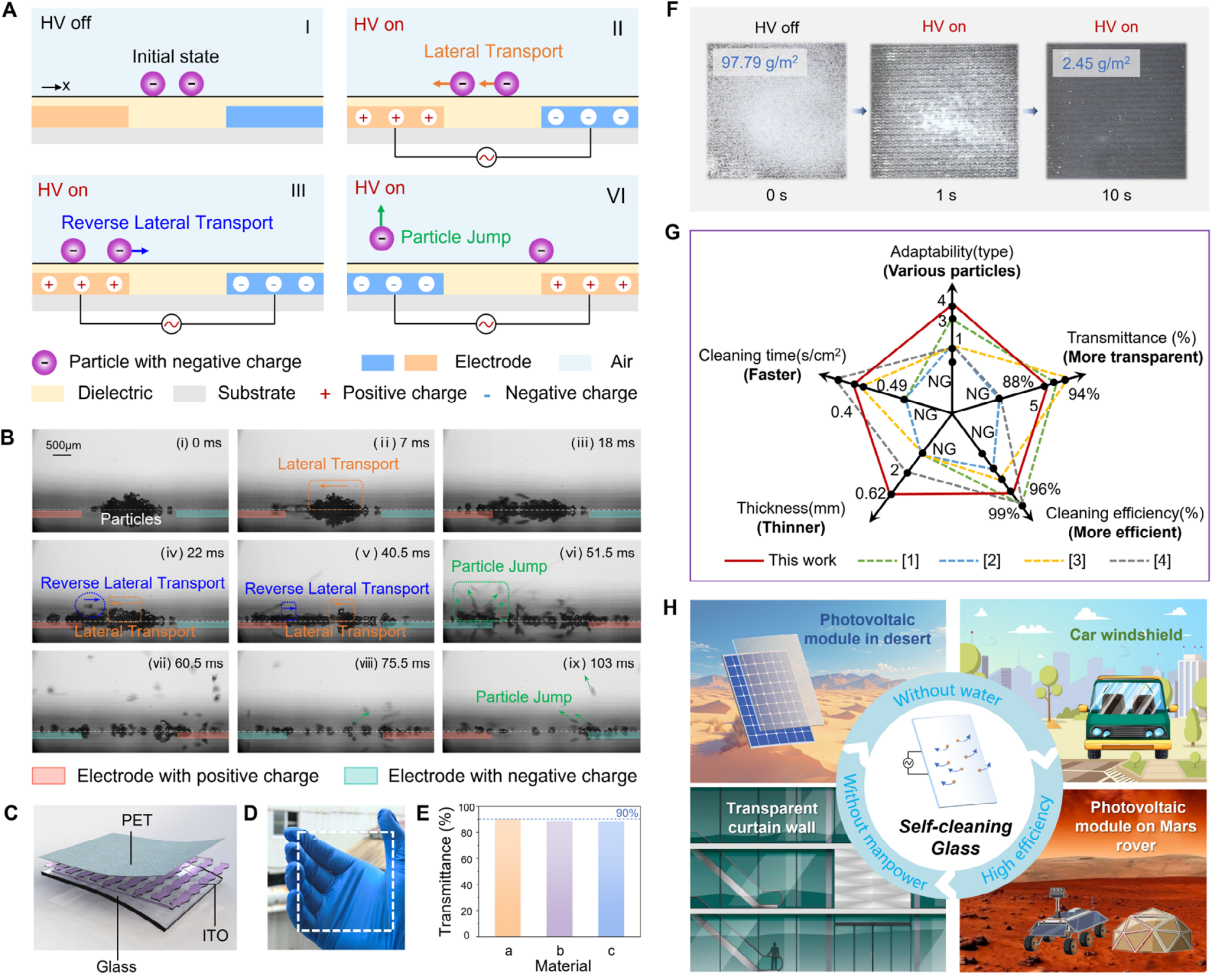
Abnormal transport and jump behaviors of charged particles and the design of transparent self‐cleaning glass. (A) Schematic diagram of abnormal transport and jump behavior of charged particles in a non‐uniform alternating electric field. (B) Photographs of abnormal transport and jump behavior of charged particles in a non‐uniform alternating electric field (Movie S1, Supporting Information). (C) Structure diagram of transparent self‐cleaning glass. (D) Photograph of transparent self‐cleaning glass. (E) The light transmittance of self‐cleaning glass. Material a is an ordinary glass. Material b is a combination of glass and ITO electrode. Material c is a combination of glass and ITO electrode, and PET film. (F) The dynamic process of surface self‐cleaning of self‐cleaning glass (Movie S2, Supporting Information). (G) The notable advantages of our self‐cleaning glass compared with other existing self‐cleaning devices driven by an electric field from five aspects. NG means that it is not given. (H) Applications of the coverable self‐cleaning glass.

Here, we report the abnormal transport and jump behaviors of charged surface particles (**Figure** **1**A), where the reverse lateral transport behavior of charged particles is observed for the first time. We build a theoretical model of particle motion and an operando visualization method to reveal the mechanism of particle transport and jump behavior. Based on this discovery, we develop a coverable self‐cleaning glass and demonstrate its strong cleaning ability for various surfaces.

## 2 Results

### 2.1 Abnormal Transport and Jump Behaviors of Charged Particles

We designed a glass with two‐electrode and placed 100 µm SiO_2_ particles between the electrodes without touching the edge of the electrode to explore the dynamic process of surface self‐cleaning behavior of particles (Figure S1, Supporting Information). And a square wave electrical signal with a voltage of 5.5 kV and a frequency of 10 Hz was applied to the electrodes. The sequential images captured by the high‐speed camera are presented in Figure 1B. After the electrical signal was applied (t = 7 ms), the particles rapidly transported laterally to the left positive electrode and deposited on it, which can be called lateral transport of particles. In this process, most of the particles transported directly across the surface, while some of the particles transported laterally in the air. Subsequently (t = 22 ms), the deposited particles around the edge of the electrode transported laterally in reverse, which can be called reverse lateral transport of particles. In addition, this abnormal particle behavior was also observed at t = 40.5 ms. This phenomenon was surprising and interesting. In traditional cognition, objects with opposite charges were attractive to each other, and those with the same charge repelled each other. In this study, an insulating polyethylene glycol terephthalate (PET) film (the dielectric constant was 3.0 and the thickness was 20 µm) was employed to isolate the electrodes and particles, which can prevent electrons from quickly transferring charges through mutual contact. Therefore, it was difficult and unacceptable to explain the reverse lateral transport behaviors of particles were caused by the principle of mutual repulsion of objects with the same charge. According to our research, there was no theoretical study to explore this phenomenon of particle transport at present, especially for the reverse lateral transport of particles.

When the electric potential of the electrode changed (t = 51.5 ms), the deposited particles on the electrode jumped suddenly. The direction and trajectory of the particles jump were like the direction of the electric field line. This phenomenon may be attributed to the comprehensive role of the high repulsive Coulomb force and dielectrophoretic force. Finally, after the end of one cycle of the electrical signal, most of the particles on the surface had been removed. These observations indicated that it was possible to achieve self‐cleaning of the surface by the abnormal transport and jump behavior of charged particles in the non‐uniform alternating electric field. And it also exhibited a great cleaning performance.

### 2.2 Design of Self‐Cleaning Glass

Based on the phenomena, we designed a coverable transparent self‐cleaning glass, as shown in Figure 1C,D. Its main structure included a glass substrate, an array of indium tin oxide (ITO) electrodes, and a polyethylene glycol terephthalate (PET) film, like a sandwich structure. After adding the ITO electrode and the PET film to the glass substrate in turn (Figure 1E), the average light transmittance of the glass decreased by 1.3% and 1.6%, respectively. Notably, the light transmittance loss of the self‐cleaning glass was concentrated in the infrared band (700–1100 nm), while the decrease in the visible band (380–700 nm) was very small (Figure S2, Supporting Information). Therefore, if self‐cleaning glass was used in photovoltaic panels, the change of light transmittance had little effect on its power generation performance. When we applied a square wave electrical signal with a voltage of 5 kV and a frequency of 10 Hz to the self‐cleaning glass, the 97.79 g m^−2^ particles on the surface rapidly decreased to 2.45 g m^−2^ within 10 s, as shown in Figure 1F. In other words, the self‐cleaning efficiency of the self‐cleaning glass surface reached 97.50%. Compared with existing self‐cleaning devices driven by electric field, our self‐cleaning glass exhibited notable advantages in terms of adaptability, glass thickness, and cleaning time (Table S1, Supporting Information). Self‐cleaning glass provided the possibility for efficient surface self‐cleaning such as photovoltaic panels in deserts, car windshields, transparent curtain wall, and photovoltaic panel of Mars probes (Figure 1H). In the face of surface self‐cleaning requirements in extreme environments, the advantages of self‐cleaning glass, such as without water, automatic, and high efficiency were very prominent and useful.

### 2.3 Mechanism Analysis

To unravel the mechanism of abnormal transport and jump behavior of charged particles observed in the experiment, we first resorted to simulating the electric field distribution on the surface by the finite element method (Figure S3, Supporting Information). At the surface, the field strength densities at the edge of the electrodes were significantly higher than that at other places. This phenomenon arose from the fact that the larger surface curvature of the electrode edge leads to a higher surface charge density. As the height from the surface gradually increased, the electric field density at the electrode edge and the electrode centers all decreased, and the difference between them gradually decreased.

Based on the above electric field distribution, theoretical modeling of particle transport and jump was conducted. Here, we summarized the main forces affecting particle motion in a non‐uniform electric field: Coulomb force ${\vec{F}}_{c}$, dielectrophoretic force $\vec{F_{dep}}$, van der Waals force $\vec{F_{vdw}}$, image force $\vec{F_{img}}$, gravitational force ${\vec{F}}_{g}$, air drag force $\vec{F_{drag}}$ and friction force ${\vec{F}}_{f}$ (see Discussion S1 in the Supporting Information for each force computation details). Consequently, the motion equation of a charged particle in the X‐Y Cartesian coordinate system based on Newton's second law of motion can be expressed as:

(1) $$\begin{array}{c} \left\{\begin{array}{c} m\;\frac{dx^{2}}{dt^{2}}=\vec{F_{c,x}}+\vec{F_{dep,x}}+\vec{F_{f}}+\vec{F_{drag,x}}\\ m\;\frac{dy^{2}}{dt^{2}}=\vec{F_{c,y}}+\vec{F_{dep,y}}+\vec{F_{drag,y}}+\vec{F_{g}}+\vec{F_{vdw}}+\vec{F_{img}}+\vec{F_{n}} \end{array}\right. \end{array}$$

We assumed that the particle charge was constant and there is no charge transfer and any loss. The core of our analysis was the initial motion behavior of a single particle from the surface in a non‐uniform electric field. Therefore, the interactions between particles were neglected and the initial state of the particle was static. In addition, the surface temperature of the self‐cleaning glass in the test chamber was maintained at 25 °C, and the humidity was maintained at 30% in this study. Therefore, the water meniscus and capillary force between the particles and the surface can be reasonably ignored.^[^ ^40^ ^]^ The force analysis of the particles was carried out from the X and Y directions respectively to obtain the mechanism of particle transport and jump comprehensively. Thus, we concluded that the condition for particle transport in a non‐uniform electric field was expressed as:

(2) $$\begin{array}{c} \vec{F_{c,x}}+\vec{F_{dep,x}}+\vec{F_{f}}+\vec{F_{drag,x}}>0 \end{array}$$

and condition for particle jump in a non‐uniform electric field can be expressed as:

(3) $$\begin{array}{c} \vec{F_{c,y}}+\vec{F_{dep,y}}+\vec{F_{drag,y}}+\vec{F_{g}}+\vec{F_{vdw}}+\vec{F_{img}}+\vec{F_{n}}>0 \end{array}$$

**Figure 2:**
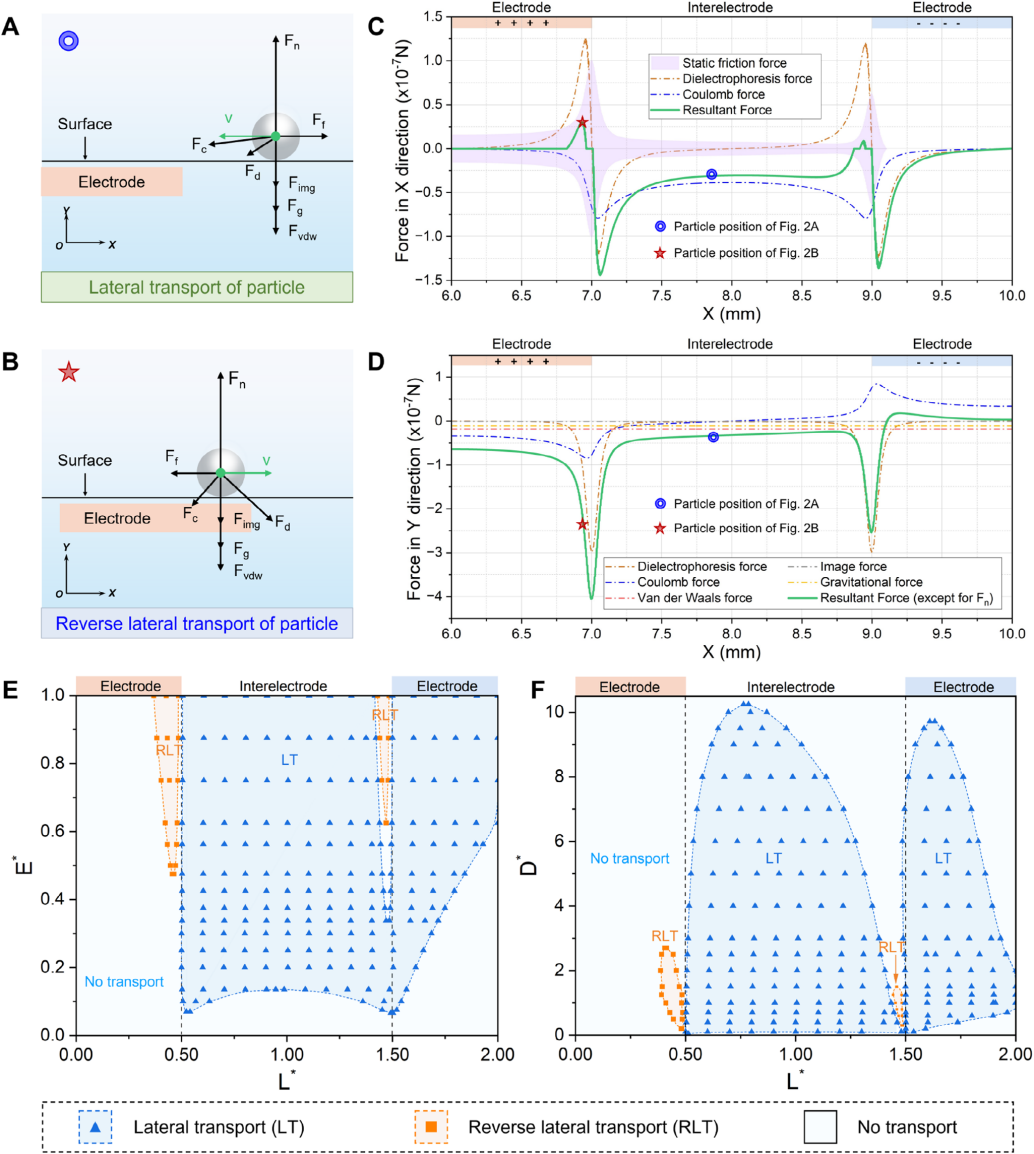
The mechanism of particle transport behaviors on the surface. (A) Force analysis of lateral transport behavior of particles. (B) Force analysis of reverse lateral transport behavior of particles. (C) Force analysis of particle transport in X direction at an applied voltage of 5.5 kV. Please note that the voltage values in this study were all peak‐to‐peak values (V_p‐p_). The average diameter of the SiO_2_ particles is 100 µm. (D) Force analysis of particle transport in the Y direction at an applied voltage of 5.5 kV. The average diameter of the SiO_2_ particles is 100 µm. (E) Phase diagram showing the behaviors of the particle transport with the variation of nondimensional electric field intensity E^*^ and nondimensional location L^*^. (F) Phase diagram showing the behaviors of the particle transport with the variation of nondimensional particle diameter D^*^ and nondimensional location L^*^.

Forces acting on particles at different locations on the surface were analyzed, as shown in **Figure** **2**. For particles at interelectrode (x = 8 mm), the resultant force in the X direction was to the left, and the downward resultant force in the Y direction was offset by the normal force *F_n_* from the surface, which leads to the lateral transport of particles. As shown in Figure 2A,C, the Coulomb force and dielectrophoretic force were the dominant force of this behavior. The resultant force to the left gradually increased as it approached the right side of the positive electrode edge. At x = 7.06 mm, the leftward resultant force exerted on the particles reached the maximum, which was −1.44 × 10^−7^ N. The peak value was mainly caused by the combination of dielectrophoretic force and Coulomb force to overcome the static friction force. However, there was an abnormal rightward resultant force in the X direction on the left side of the positive electrode edge, which can lead to reverse lateral transport of particles. And its peak was at x = 6.93 mm, up to 2.99 × 10^−8^ N. This abnormal phenomenon can be attributed to dielectrophoretic force on the particles was much larger than the resistance composed of friction and Coulomb force due to the high electric field density gradient and dipole moment induced in the dielectric particles, as shown in Figure 2B. It can be seen that the Coulomb force plays a dual role of driving force and resistance in the self‐cleaning process.

For particles at the edge (x = 7 and 9 mm) and the central location (x = 8 and 10 mm) of electrode, the resultant force exerting on the particles in the X direction was 0 N due to the limitation of the static friction force. And the resultant force in the Y direction was also 0 N due to the limitation of the normal force *F_n_* from the surface (Figure 2D). Therefore, the particles were theoretically stationary at these locations, which was called the particle stagnation zone. This observed excellent agreement with the results of Sun et al.^[^ ^41^ ^]^ and Gu et al.^[^ ^31^ ^]^ And we also observed that the particles were trapped at the edge of the electrode (particle stagnation zone) during the experiment, as shown in **Figure** **5**A.

To further reveal the mechanism of particle transport on the surface, we define the nondimensional electric field intensity E^*^ by nondimensionalizing the electric field intensity (E) using the breakdown electric field value in the air (*E_0_* = 3 × 10^6^ V m^−1^) as $E^{*}=\frac{E}{E_{0}}$.And a nondimensional location ($L^{*}=\frac{X}{L}$) was defined to describe the relationship between the particle location and electrode position, where L was the width of the electrode and also the width of the interelectrode. Based on the particle motion behaviors, we proposed that the behavior of particles moving toward the positive electrode was called lateral transport of particles (LT). And the behavior of particles moving toward the negative electrode was called reverse lateral transport of particles (RLT). Figure 2E presents the phase diagram of the behaviors of the particle transport with the variation of nondimensional electric field intensity E^*^ and nondimensional location L^*^. At low electric field density, there was no particle transport at low electric field density due to the small Coulomb force and dielectrophoretic force. The blue‐shaded region of LT first began to appear near the edge of the electrode and gradually expanded with the electric field density. At E*= 0.475 and E*= 1.472, the orange‐shaded region of RLT began to be observed at the right edge of the positive electrode and the interelectrode, respectively. The asymmetry of these regions was mainly caused by the variable roles of the Coulomb force as resistance and driving force at different locations.

Nondimensional particle diameter ($D^{*}=\frac{d}{d_{100}}$) was defined to investigate the behaviors of the particle transport with the variation of nondimensional particle diameter D^*^ and nondimensional location L*, as shown in Figure 2F. Here, the particle diameter of 100 µm (*d*_100_) was used as a baseline to nondimensionalize the particle diameter. It can be seen that there was no transport behavior for particles with fine particles (D*<0.003) and large particles (D*>10.24). The limitation of the static friction force caused by van der Waals force and gravitational force was the reason why fine particles and large particles were not driven respectively. At the edge of the positive electrode and the right side of the interelectrode, the particles were mainly limited by the static friction force caused by the combined effects of the gravity, the coulomb force, and the dielectrophoretic force in the Y direction which increased rapidly with the particle size. Thus, there was only the behavior of LT and no RLT for medium‐sized particles (2.7<D*<11.7).

**Figure 3:**
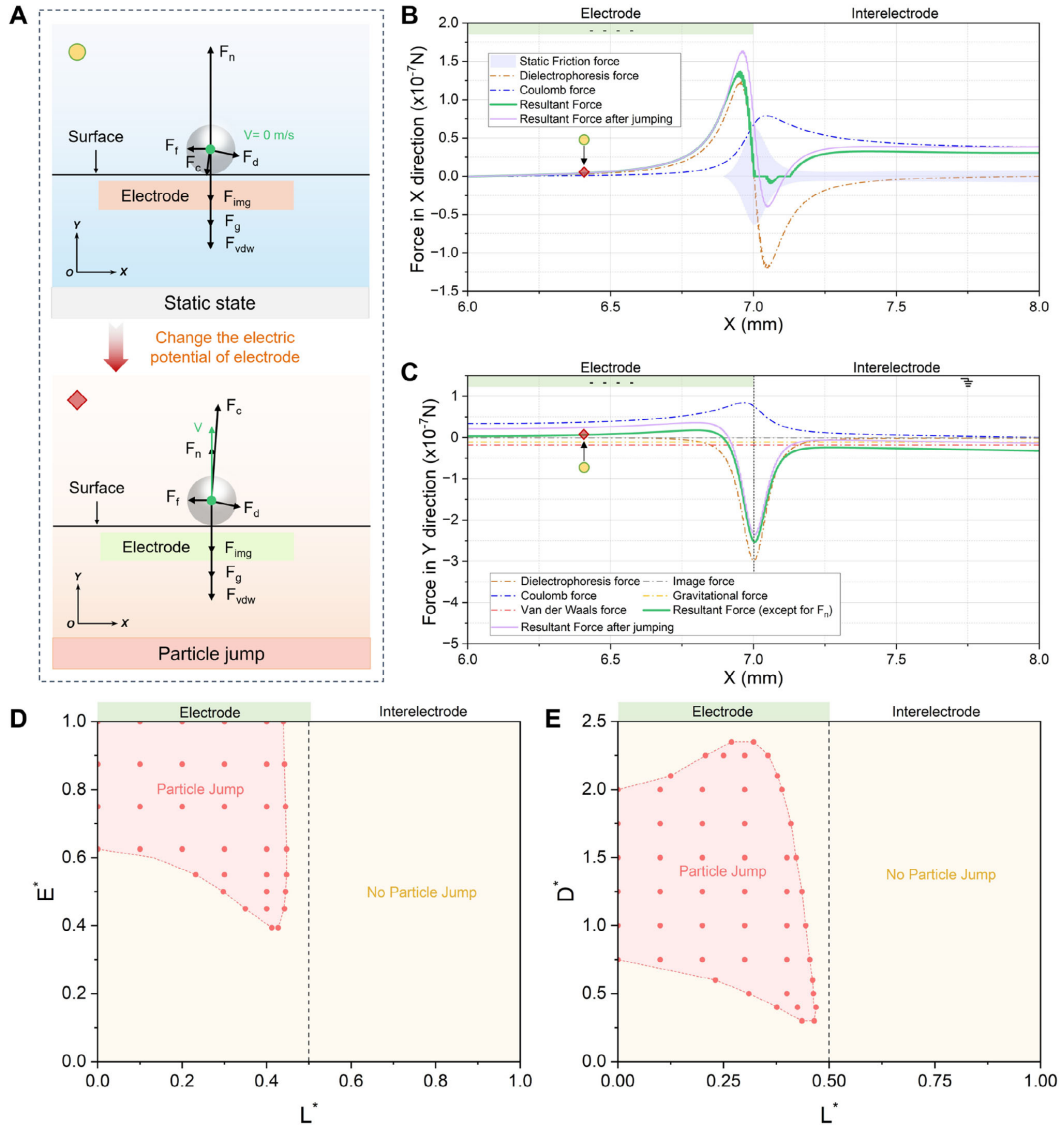
The mechanism of particle jump behavior on the surface. (A) Force analysis of particle jump after the electric potential of the electrode change. (B) Force analysis of particle jump in X direction at an applied voltage of 5.5 kV. The average diameter of the SiO_2_ particles is 100 µm. (C) Force analysis of particle jump in Y direction at an applied voltage of 5.5 kV. The average diameter of the SiO_2_ particles is 100 µm. (D) Phase diagram showing the behaviors of the particle jump with the variation of nondimensional electric field intensity E^*^ and nondimensional location L^*^. (E) Phase diagram showing the behaviors of the particle jump with the variation of nondimensional particle diameter D^*^ and nondimensional location L^*^.

In addition to the particle transport, the particle jump is also a typical behavior in a non‐uniform alternating electric field. As shown in **Figure** **3**A, the change of the electric potential of the electrode significantly affected the forces acting on the particles. The deposited particles caused by the particle transport on the positive electrode began to jump by an upward resultant force in the Y direction when the electrode was changed into a negative electrode, as illustrated in Figure 3B,C. In this process, the Coulomb force was the dominant force. The direction of particle jump at the center of the electrode (x = 6 mm) was almost vertical upward, while the particles near the edge of the electrode (x = 6.75 mm) showed a certain angle of jump due to the influence of dielectrophoretic force. However, the edge of the electrodes (x = 7 mm) was still the particle stagnation zone. It can be seen that the potential regulation of the electrode cannot effectively remove the particles trapped at the edge of the electrode (particle stagnation zone) in a non‐uniform alternating electric field. After particle jumping, the resultant force of the particle in the Y direction in the central region of the electrode (x = 6–6.89 mm) increased by ≈2.45 times on average, which means that the particles in this region will be further accelerated to jump off the surface after jumping.

The effects of electric field density and particle size on the behaviors of particle jump are shown in Figure 3D,E. At E*= 0.393, the jump behavior of particles began to appear at the left location of the electrode edge (L*= 0.42) due to stronger Coulomb force and gradually expanded to the center of the negative electrode with the electric field density. When the electric field strength E* was 0.625, the particle jump region in the non‐uniform electric field reached the maximum value, and the particles can jump in the region of L*= 0.0–0.447. After that, even if the electric field strength continued to increase, the region of the particle jump cannot be expanded. In addition, it can be seen from the phase diagram that the particles at the edge of the electrode (L*= 0.447–0.45) cannot jump regardless of how to increase the electric field intensity. The reason was that the strong electric field intensity gradient in this region led to a large Y component of the dielectrophoretic force, which limited the jump behavior of the particles.

There was no jump behavior for particles with fine particles (D*<0.3) and large particles (D*>2.35), as shown in Figure 3E. This was mainly because fine particles were dominated by van der Waals force, and large particles were dominated by gravitational force, resulting in particles adhering to the surface and difficult to jump. When the particle size D* was greater than 0.3 and less than 0.75, the region of particle jump behavior gradually expanded to the center of the electrode with the increase of particle size. When the particle size D* greater than 0.75, the region of particle jump behavior (red background) shrank with the increase of particle size. This was attributed to the fact that the dielectrophoretic force and gravitational force, which were the main forces hindering the particle jump, grew faster with the particle size (∝*d^3^*) than the Coulomb force which was the only driving force of the particle jump.

According to the above discussion, it can be seen that the lateral transport behavior of particles mainly occurred in the interelectrode region. The reverse lateral transport behavior of particles mainly occurred in the edge region of the electrode. And the jump behavior of particles mainly occurred in the electrode center region. It can be further concluded that the threshold voltage required for the particles to begin to transport (E*= 0.06) was much lower than that for the particle jump (E*= 0.393). Therefore, the particle transport phenomenon during the self‐cleaning process was easier to be observed than the particle jump in theory, especially for cleaning fine particles at low applied voltage. This can well explain the rearrangement phenomenon of 33.49 µm particles observed by Gu et al.^[^ ^29^ ^]^ at the moment of applying an electrical signal with a voltage of 2.4 kV. In practical applications, the transport and jump behavior of particles can be enhanced by increasing the voltage of the electrical signal. In addition, it can be considered to further improve the self‐cleaning performance by optimizing the electrode configuration to eliminate the particle stagnation zone, strengthening the transport behavior of the particles to increase the number of particles deposited in the center of the electrode.

### 2.4 Dynamic Process Analysis of a Single Charged Particle

**Figure 4:**
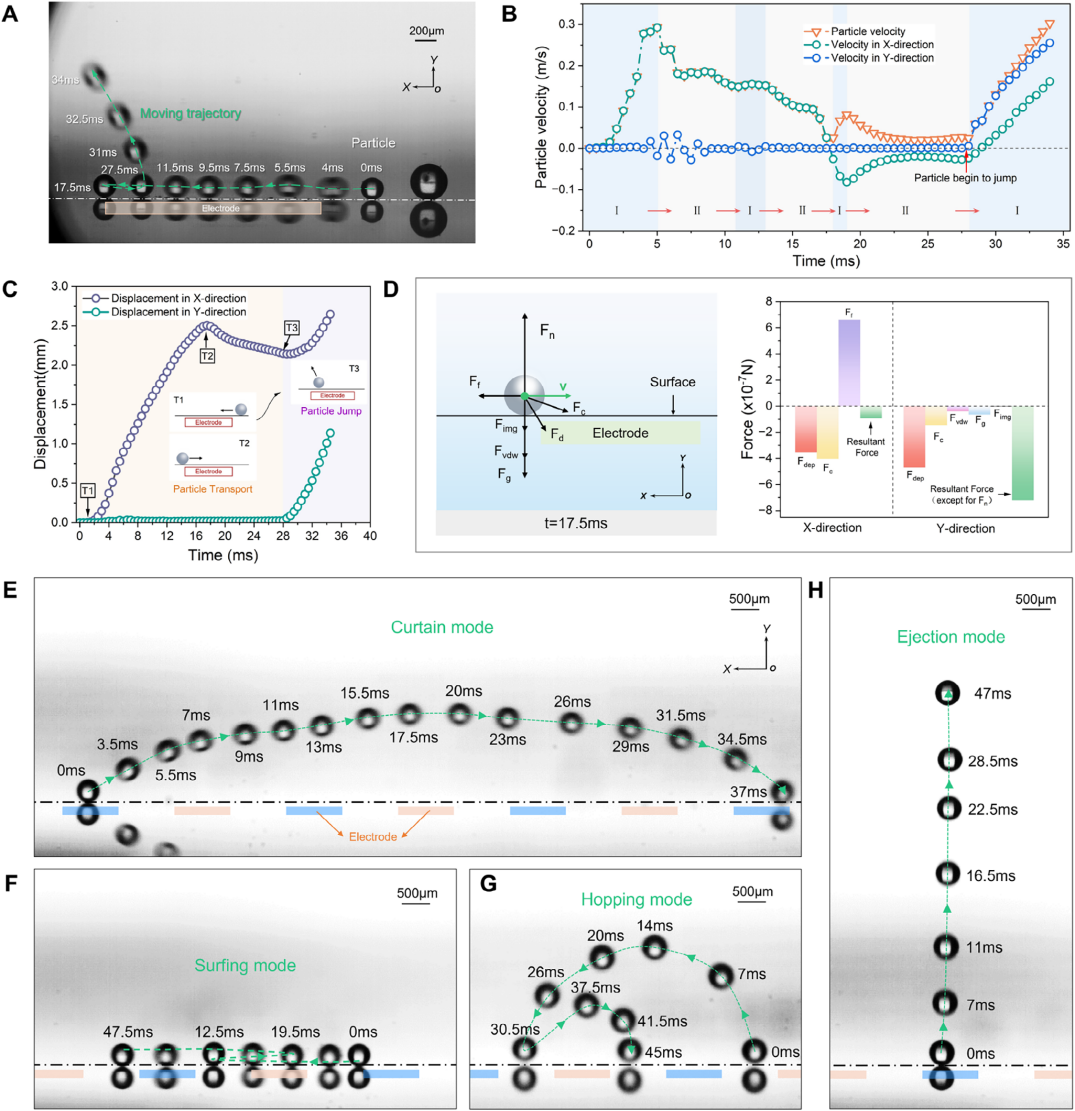
Dynamic process analysis of a single charged particle transport and jump behaviors. (A) Dynamic behavior of a single particle transport (Movie S3, Supporting Information). The signal of applied voltage is a single‐phase square wave with a voltage of 5.5 kV and a frequency of 5 Hz. The diameter of the moving Polymethyl Methacrylate (PMMA) particle is ≈221 µm. (B) Particle velocity with time. Region I represents the acceleration region of particle motion. Region II represents the acceleration region of particle motion. (C) Particle displacement with time. The light orange background represents that the particles are in the state of particle transport. The light purple background represents that the particles are in the state of particle jump. (D) Force analysis of the change of particle transport direction at t = 17.5 ms. (E) Curtain mode (Movie S5, Supporting Information). The signal of applied voltage is a single‐phase square wave with a voltage of 4.5 kV and a frequency of 15 Hz. (F) Surfing mode (Movie S6, Supporting Information). The signal of applied voltage is a single‐phase square wave with a voltage of 4.5 kV and a frequency of 15 Hz. (G) Hopping mode (Movie S7, Supporting Information). The signal of applied voltage is a single‐phase square wave with a voltage of 4.0 kV and a frequency of 10 Hz. (H) Ejection mode (Movie S8, Supporting Information). The signal of applied voltage is a single‐phase square wave with a voltage of 4.0 kV and a frequency of 10 Hz.

To gain a deeper understanding of the mechanism of the particle transport and jump behavior, we used a high‐speed camera to capture the transport and jump behaviors of a single particle, as illustrated in **Figure** **4**A. And the velocity and displacement of the particles with time during the process of particle transport are shown in Figure 4B,C. Initially (t = 0 s), a 220 µm particle was placed at the interelectrode region ≈0.4862 mm from the edge of the electrode and then had a typical lateral transport behavior which was continuously accelerated by the leftward resultant force of the LT region in the non‐uniform electric field. The transport velocity of the particles reached a maximum of 0.29 m s^−1^ at t = 5 ms. At the stage of t = 5–6.5 ms, the particle was rapidly decelerated by the rightward resultant force of the RLT region. Note that, the fluctuation of the velocity in the Y direction of the particle at t = 5–7.5 ms was mainly due to the uneven surface caused by the adhesion of ultrafine particles. The slight acceleration behavior of the particle at t = 11.5–13 ms can be observed, which was attributed to the leftward resultant force of the RLT region.

After t = 16.5 ms, the particle began to leave the left edge of the electrode and was subjected to the rightward resultant force of the LT region. Therefore, the velocity of the particle slowed down rapidly and began to move to the right at t = 17.5 ms. Figure 4D represents the force analysis of the change of particle transport direction at t = 17.5 ms. It can be seen that the dielectrophoretic force and Coulomb force were the dominant forces in this process. The reason for the deceleration of particles at t = 19–27.5 ms was the same as that of particle acceleration at t = 11.5–13 ms. At t = 27.5 ms, the particle on the electrode was driven by a strong Coulomb force in the Y direction and began to jump, which was caused by the change of the electric potential of the electrode. The particle was also affected by the leftward resultant force of the RLT region in this process. Therefore, the motion direction of the particle was to the upper left. The speed of particle jump had reached 0.30 m s^−1^ at t = 34 ms, as shown in Figure 4B. The experimental results prove the validity of the phase diagram in section 3.2. In addition, another typical behavior here was also observed that a single particle jumped directly in the process of particle transport (Figure S4 and Movie S4, Supporting Information).

The behaviors of charged particles after jump were affected by many factors, such as the electric field, flow field, position and velocity of particle jump, and collision between particles and the surface. Thus, they were complex and variable. Here, we further explored various motion modes of a single particle after a particle jump, as shown in Figure 4E–H. During the experiments, four typical motion modes were recorded by the high‐speed camera, which were curtain mode, ejection mode, surfing mode, and hopping mode.

As illustrated in Figure 4E, the particle of the curtain mode floated above the surface, and continued to move forward in the X direction in the process of continuous acceleration and deceleration. The average floating height and average velocity of the particle at the stage of t = 7–31.5 ms were 1.31 mm and 0.35 m s^−1^. This floating behavior can be simply explained as a continuous lifting of the resultant force in the Y direction whenever the particles fell. Figure 4F shows another typical surfing mode of a particle. The particle rolled repeatedly along the surface in this mode. The LT and RLT region for the particles lead to the occurrence of this mode. The reason why the particle had no jump behavior may be that the insufficient charge of the particle led to a smaller Coulomb force in the Y direction. Figure 4H shows an ejection mode of a particle that has never been reported before. The particle of the ejection mode was ejected directly from the surface almost vertically. At t = 12 ms, the particle reached the maximum speed of 0.25 m s^−1^. And the maximum height of the particles can exceed 6 mm. The hopping mode of the particle is represented in Figure 4G. The characteristic of this mode was that the whole motion process was composed of a series of successive jump behaviors. In this process, the maximum height was 1.84 mm at t = 14 ms. Compared with the curtain mode, ejection mode, and surfing mode, the hopping mode was unstable and vulnerable to small disturbances to change the jump direction and particle velocity. The occurrence of the hopping mode was likely to be caused by the continuous combined effects of particle transport and particle jump. It should be noted that according to our experimental observation, there were multiple transitions between various modes in the motion of a single particle. The various motion modes of particles after jump behavior provide a theoretical possibility for the directional transport, separation,^[^ ^42^ ^]^ in situ utilization^[^ ^43^ ^]^ of particles, and rapid self‐cleaning of the surface.

### 2.5 Application of Coverable Self‐Cleaning Glass

**Figure 5:**
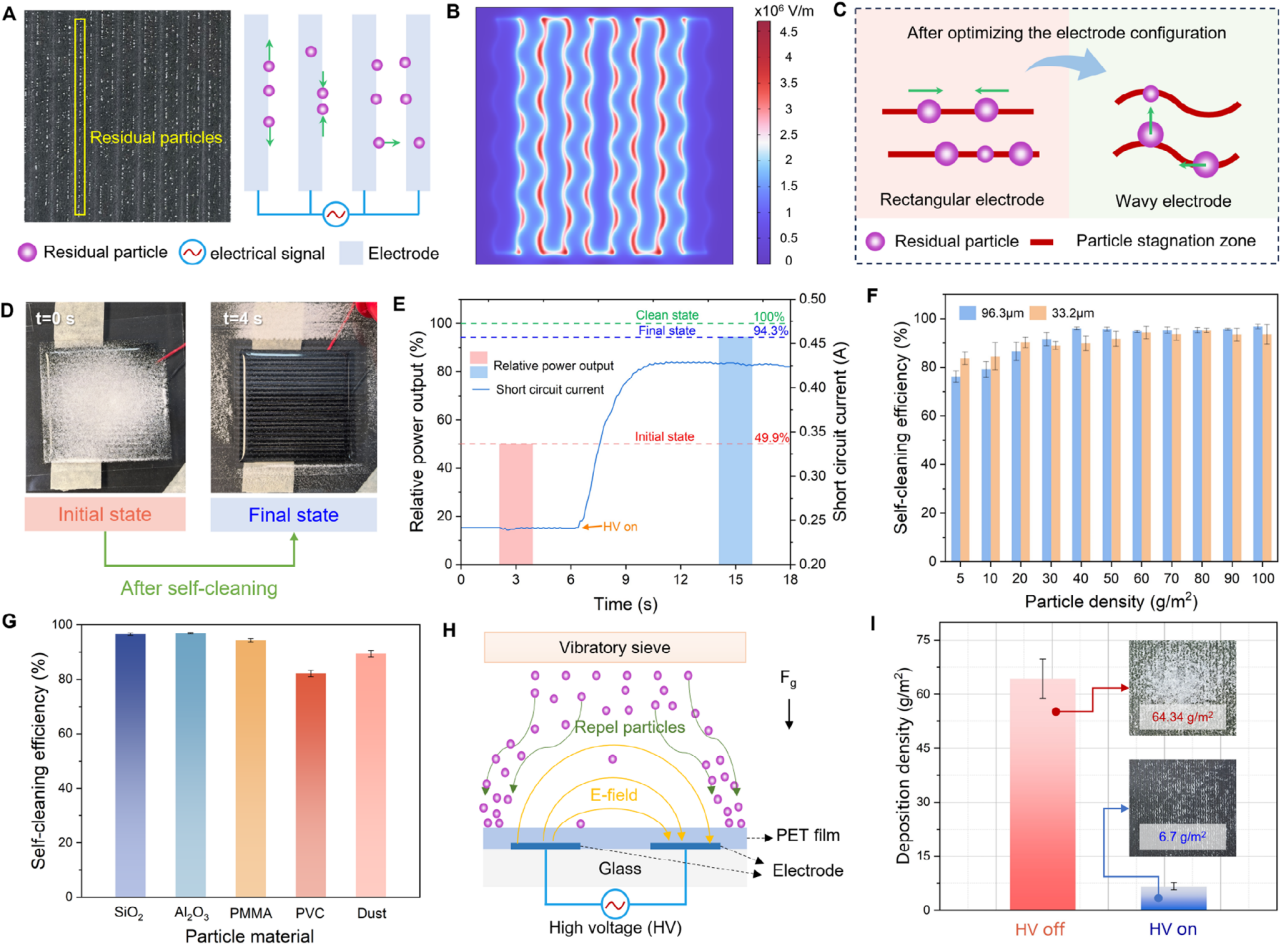
Application of coverable transparent self‐cleaning glass. (A) Residual particles at the edge of the rectangular electrode after the self‐cleaning process. (B) Electric field distribution on the surface of self‐cleaning glass with wavy electrodes. (C) Analysis of particle behavior in the particle stagnation zone after optimizing the electrode configuration. (D) Surface self‐cleaning demonstration on a lab‐scale photovoltaic panel (Movie S9, Supporting Information). (E) Power output and real‐time short circuit current response of a lab‐scale photovoltaic panel during the surface self‐cleaning process. (F) Surface self‐cleaning efficiency under various particle densities. (G) Surface self‐cleaning efficiency under various particle materials. The dust was collected from the surface of photovoltaic panels in the Nei Mongol Autonomous Region of China (Figure S9, Supporting Information). (H) Schematic of self‐cleaning glass to prevent charged particles in the air from depositing on the surface. (I) The effect of self‐cleaning glass on preventing charged particles in the air from depositing on the surface.

Based on the mechanism of particle transport and jump behavior, how to achieve efficient surface self‐cleaning of self‐cleaning glass was a very important issue that we were concerned about. We first selected the self‐cleaning glass with the rectangular ITO electrode configuration for the surface self‐cleaning test. It was found that most of the particles can leave the surface after applying an electrical signal. However, there were still some residual particles on the self‐cleaning glass surface, which were mainly distributed in a linear shape near the edge of the electrode, as shown in Figure 5A. It is worth noting that these residual particles were not always in a stationary state. During the experiment, it can be clearly observed that some particles repeatedly moved along the edge of the electrode (linear particle stagnation zone) and sometimes collided with each other. Some particles can suddenly break away from the stagnation zone after the collision, and then transport or jump. This interesting phenomenon may be due to the fact that the local electric field between the residual particles in the non‐uniform alternating electric field was significantly enhanced when the square wave electrical signal reached a high level, resulting in mutual repulsion between the particles due to the Coulomb force. Therefore, the particles obtained a certain initial kinetic energy. However, under the limitation of the linear particle stagnation zone, they can only move along the edge of the electrode. After the self‐cleaning process, no matter how the electrical signal was applied, most of the linear residual particles in the stagnation zone cannot be driven off the surface. Although these residual particles were little (≈4.13% of the initial particle deposition), there are still problems that cannot be ignored. For example, for photovoltaic panels in the desert, these non‐uniform linear particle chains may produce local hot spots, which may cause damage to the entire photovoltaic panel.

To solve the above problem, we designed a self‐cleaning glass with a wavy electrode configuration (Figure 1C). The electric field distribution on the surface of self‐cleaning glass with wavy electrodes can be seen in Figure 5B. The high electric field intensity regions were mainly located at the convex edge of the electrode, and the maximum value can reach 2.74 × 10^6^ V m^−1^. Compared with the rectangular electrode configuration, the residual particles of the self‐cleaning glass with the wavy electrode configuration were significantly more uniform and dispersed (Figure S5, Supporting Information). Combined with the electric field distribution of the surface, it can be inferred that there are two reasons for the above phenomenon. First, the wavy electrode had a certain radius, which caused the particle stagnation zone at the edge of the electrode to be curved. Compared with the linear particle stagnation zone, it was easier for the particles in the curved particle stagnation zone to leave by particle inertia when they moved along the stagnation zone, as shown in Figure 5C. Second, there are many high electric field intensity regions on the surface, which can promote the collision of residual particles (Figure S6, Supporting Information), resulting in the dispersed distribution of residual particles.

Due to the significant economic benefits of cleaning of photovoltaic panels, we use self‐cleaning glass to cover the photovoltaic panels to verify their cleaning ability (Figure 5D). After applying a square wave electrical signal with the voltage of 5 kV and the frequency of 10 Hz, the self‐cleaning photovoltaic panel contaminated by 100 g m^−2^ particles completed surface self‐cleaning within 4 s. Figure 5E shows the relative output power and short circuit current changes during the self‐cleaning process of the photovoltaic panel. Based on the output power of the clean photovoltaic panel, the output power of the contaminated photovoltaic panel was reduced by 50.1%, and the short circuit current was 0.242 mA. After the photovoltaic panel began to self‐clean the surface (t = 6.6 s), the deposited particles continued to leave the surface driven by the electric field, and the short circuit current of the photovoltaic panel gradually increased. The short circuit current of the photovoltaic panel after the surface self‐cleaning process (t = 11.2 s) was stable at 0.429 mA. The relative output power was increased by 88.2%, reaching 94.3%.

The surface self‐cleaning efficiency of the photovoltaic panel under various particle densities is further analyzed, as shown Figure 5F. When the particle deposition density was 5 g m^−2^, the self‐cleaning efficiency of 96.3 and 33.2 µm particles was 76.17% and 83.74%, respectively. With the increase of particle deposition density, the surface self‐cleaning efficiency of the two particle sizes gradually increased and tended to be stable. It can be seen that even at high particle deposition density, the surface self‐cleaning performance of the photovoltaic panel was still excellent. For example, when the particle deposition density was 100 g m^−2^, the surface self‐cleaning efficiency of 96.3 and 33.2 µm particles was 96.83% and 93.54%, respectively. Compared with high particle deposition density, the self‐cleaning efficiency of the photovoltaic panel was relatively low at low particle deposition density, which may be related to the insufficient collision between particles.

Figure 5G shows the surface self‐cleaning efficiency of a photovoltaic panel under various particle materials. Among them, SiO_2_ particles and Al_2_O_3_ particles were inorganic particles, Polymethyl Methacrylate (PMMA) particles and Polyvinyl chloride (PVC) particles were organic particles. The self‐cleaning efficiency of the above four particles on the photovoltaic surface decreased in the following sequence: Al_2_O_3_ particles (96.92%)>SiO_2_ particles (96.56%)>PMMA particles (94.38%)>PVC particles (94.38%). The density of Al_2_O_3_ particles was higher than that of the other three particles, which was theoretically more limited by gravity. However, the self‐cleaning efficiency of Al_2_O_3_ particles was the highest, which can be attributed to the following two aspects. First of all, Al_2_O_3_ particles had a high dielectric constant (ε = 9.8), resulting in a higher dielectrophoretic force in the electric field, which will enhance the transport behavior of particles in the interelectrode region. Second, the shape of Al_2_O_3_ particles tended to be more spherical, which will result in a lower friction force on the particles. Compared with the other three particles, PVC particles had the lowest density and were theoretically least limited by gravity. However, PVC particles have the lowest self‐cleaning efficiency, which may be attributed to the strong polar C─Cl bonds in PVC particles increasing the van der Waals force between particles and the surface. In addition, the roughness of PVC particles was larger, and the friction force between particles and the surface was higher. Nevertheless, the self‐cleaning glass designed in this paper shows an excellent self‐cleaning effect on organic particles and inorganic particles. To further verify the self‐cleaning performance of self‐cleaning glass on dust, we first collected dust on the surface of photovoltaic panels in the Nei Mongol Autonomous Region of China (Figure S9A, Supporting Information). The average diameter of the dust particles was 28.1 µm (Figure S9B, Supporting Information). Surface self‐cleaning efficiency of self‐cleaning glass under dust pollution condition can reach 89.43%, as shown in Figure S10 and Movie S10, Supporting Information).

In addition to the surface self‐cleaning ability, the self‐cleaning glass can also prevent moving particles in the air from depositing on the clean surface after applying an alternating voltage, as shown in Figure 5H,I. The particle deposition density on the surface of self‐cleaning glass without an electrical signal was 64.34 g m^−2^. However, after applying a square wave electrical signal with a voltage of 5 kV and a frequency of 10 Hz, the amount of particle deposition on the self‐cleaning surface was only 6.7 g m^−2^, which was reduced by 89.59%. We defined this phenomenon as the particle shielding effect of self‐cleaning glass. The reason for this was that the particles in the alternating electric field were subjected to the Coulomb force, which directly repelled some of the moving particles in the air to move along the electric field line, which prevented moving particles from depositing on the surface. At the same time, due to the action of dielectrophoretic force and Coulomb force, the surface also attracted some moving particles to accelerate the movement to the surface. These particles will continue to transport and jump on the surface until they finally leave the surface. For photovoltaic panels in the desert, they were often affected by dusty weather such as sandstorms. The particle shielding effect of self‐cleaning glass can effectively reduce the deposition of particles in the air to the surface to achieve sustainable operation of photovoltaic panels.

## 3 Discussion

In summary, we report a newly observed abnormal transport and jump behavior of charged particles in a non‐uniform alternating electric field and design a transparent self‐cleaning glass based on this phenomenon. The theoretical modeling of transport and jump behavior of charged particles was derived and observed by operando visualization experiments. The self‐cleaning efficiency of self‐cleaning glass can reach more than 95% within 10 s. The self‐cleaning glass can also achieve self‐cleaning of organic and inorganic particles.

## 4 Experimental Section

### Visualization of the Dynamic Process of Surface Self‐Cleaning and a Single Particle Transport and Jump

To explore the motion behavior of micron particles in the electric field, an operando visualization experimental system was designed, which mainly consisted of five main sections: humidity control section, power supply section, test chamber section, surface temperature control section, and operando visualization section (See Figure S7, Supporting Information for more experimental details). Before the start of the experiments, the particle samples were placed in a vacuum drying chamber (LC‐DZF‐6050AB, LICHEN, China) and dried for 12 h to avoid the influence of adsorbed moisture in the particles. And the dried particles were fully shaken in a stainless‐steel cup for 2 min, which can fully charge the particles through the friction and collision between the particles and the cup surface. Here, SiO_2_ particles with an average diameter of 100 µm were selected as simulated contaminant for exploring the dynamic process of surface self‐cleaning. To more clearly track the motion behavior of particles, PMMA particles with lighter weight and lower dielectric constant were selected for the observation of a single particle transport and jump. During the experiments, the surface temperature of the self‐cleaning glass in the test chamber was maintained at 25 °C, and the humidity was maintained at 30% by adjusting the humidity control section and the surface temperature control section. It is worth noting that the residual particles on the surface after the experiments should be gently removed with a brush to avoid particles scratching the surface.

### Fabrication of Self‐Cleaning Glass

The typical configuration of self‐cleaning glass was mainly composed of substrate, electrode, and dielectric layer. In this study, quartz glass, indium tin oxide (ITO), and polyethylene glycol terephthalate (PET) film were selected as the substrate, electrode, and dielectric layer, respectively. The thickness, length, and width of quartz glass were 0.62, 90, and 70 mm respectively. For a glass with two‐electrode, the ITO electrodes with a thickness of 185 nm, a width of 2 mm were covered on the quartz glass by laser etching. And the interelectrode width was also 2 mm. For self‐cleaning glass with multi‐electrode, the thickness and the width of the electrode and interelectrode width were 185 nm, 1 mm, and 1 mm, respectively. An insulating PET film with 20 µm thickness was covered on ITO electrodes to prevent electrical breakdown.

### Experiments on Self‐Cleaning Efficiency

Particles were uniformly deposited on the surface of the self‐cleaning glass through a mesh sieve to simulate the deposition of contaminant on photovoltaic panels in the desert. A high‐precision electronic analytical balance (LC‐FA2004, LICHEN, China) with a least count of 0.1 mg was selected for particle mass measurement. To precisely control the deposition of particles in the self‐cleaning area of the glass, a hollow acrylic plate was designed to fit the glass surface to limit the particle deposition position. Therefore, the self‐cleaning efficiency of glass at different particle densities can be calculated by the variation of particle mass.

### Light Transmittance and the Lab‐Scale Self‐Cleaning Photovoltaic Panel Monitoring

The light transmittance of the self‐cleaning glass was obtained with a desktop spectral transmittance measurement instrument (Filmeasure2150, Beijing AOPTEK Technology, China). The self‐cleaning photovoltaic panel was composed of a solar cell with a length and width of 45 mm (ZD45 × 45, ZDSOLAR, China) and self‐cleaning glass. The open circuit voltage (V_oc_) of the photovoltaic module was measured by a multimeter (VC890C, VICTOR Technology, China). The short circuit current (I_sc_) of the photovoltaic module was monitored in real time by an electrochemical workstation (CHI660E, Shanghai Chinstruments, China).

### Image and Data Processing

To extract particle velocity and position from the high‐speed movie, a MATLAB (MathWorks) code was developed to track the centroid of the particle. Origin 2024 and Image J were employed for image processing and statistical analysis.

## Conflict of Interest

The authors declare no conflict of interest.

## Supporting information



Supporting Information

Supplemental Movie 1

Supplemental Movie 2

Supplemental Movie 3

Supplemental Movie 4

Supplemental Movie 5

Supplemental Movie 6

Supplemental Movie 7

Supplemental Movie 8

Supplemental Movie 9

Supplemental Movie 10

## Data Availability

All data needed to evaluate the conclusions in the paper are present in the paper and/or the Supplementary Materials.
