# Supplementary material for: Coverable Self‐Cleaning Glass via Abnormal Transport and Jump of Charged Particles
Source: Adv Sci (Weinh). 2025 Jul 26;12(39):e09404. doi: 10.1002/advs.202509404 (PMC12533384; doi:10.1002/advs.202509404)
Supplement: Supplementary file 1 — Supporting Information [file ADVS-12-e09404-s003.docx]

Supplementary Materials

# Coverable self-cleaning glass via abnormal transport and jump of charged particles

Meng Yang^1^, Conglin Li^1^, Wei Tang^2^, Wenchao Gao^3^, Weiguo Weng^4^, Yingchun Wu^1^, Yifan Wang^1,5,*^, Chenghang Zheng^1,5,6,*^, Xiang Gao^1,5,6, *^

*1: State Key Laboratory of Clean Energy Utilization, State Environmental Protection Engineering Center for Coal-Fired Air Pollution Control, Zhejiang University, Hangzhou 310027, People’s Republic of China*

*2: State Key Laboratory of Fluid Power and Mechatronic Systems, Zhejiang University, Hangzhou 310027, China*

*3: Beijing Institute of Nanoenergy and Nanosystems, Chinese Academy of Sciences, Beijing 100083, People's Republic of China*

*4: Jiaxing Research Institute, Zhejiang University, Jiaxing 314031, China*

*5: Institute of Carbon Neutrality, Zhejiang University, Hangzhou 310027, People’s Republic of China*

*6: Zhejiang Baima Lake Laboratory Co., Ltd., Hangzhou 310051, People’s Republic of China*

* Corresponding author: Dr. Yifan WANG, Prof. Chenghang ZHENG and Prof. Xiang GAO.

State Key Lab of Clean Energy Utilization,

Institute for Thermal Power Engineering

Zhejiang University

Hangzhou 310027

P.R. China

E-mail addresses: yfw1@zju.edu.cn (Y. Wang), zhengch2003@zju.edu.cn (C. Zheng), xgao1@zju.edu.cn (X. Gao)

Tel & Fax: +86-571-87953129 (C. Zheng), +86-571-87951335 (X. Gao)

**This PDF file includes:**

**Supplementary Fig. S1**. Initial location of SiO_2_ particles on the glass with two-electrode

**Supplementary Fig. S2**. The light transmittance of the self-cleaning glass and ordinary glass

**Supplementary Fig. S3**. Electric field distribution above surface of the glass with two-electrode

**Supplementary Fig. S4**. A single particle jumped directly in the process of particle transport

**Supplementary Fig. S5**. Residual particles at the edge of the wavy electrode after self-cleaning process

**Supplementary Fig. S6**. Dynamic behavior of collision between residual particles during self-cleaning process

**Supplementary Fig. S7**. Schematic of the operando visualization experimental system

**Supplementary Fig. S8**. Force analysis of particles in non-uniform alternating electric field

**Supplementary Fig. S9.** Dust from the surface of photovoltaic panels in Nei Mongol Autonomous Region of China

**Supplementary Fig. S10**. Surface self-cleaning demonstration under dust pollution condition

**Supplementary Discussion 1.** Details of the main forces affecting particle motion in non-uniform electric field

**Supplementary Table S1**. The notable advantages of our self-cleaning glass compared with other existing self-cleaning device from six aspects

**Supplementary References** (1 to 10)

**Other Supplementary Materials for this manuscript include the following:**

**Supplementary Movie S1**. Abnormal transport and jump behavior of charged particles in non-uniform alternating electric field

**Supplementary Movie S2**. The dynamic process of surface self-cleaning of self-cleaning glass

**Supplementary Movie S3**. The transport and jump behavior of a single particle in non-uniform alternating electric field

**Supplementary Movie S4**. A single particle jumped directly in the process of particle transport in non-uniform alternating electric field

**Supplementary Movie S5**. Curtain mode of a single particle in non-uniform alternating electric field

**Supplementary Movie S6**. Surfing mode of a single particle in non-uniform alternating electric field

**Supplementary Movie S7**. Hopping mode of a single particle in non-uniform alternating electric field

**Supplementary Movie S8**. Ejection mode of a single particle in non-uniform alternating electric field

**Supplementary Movie S9**. Surface self-cleaning demonstration on photovoltaic panels

**Supplementary Movie S10**. Surface self-cleaning demonstration under dust pollution condition

**Supplementary Figures**

**
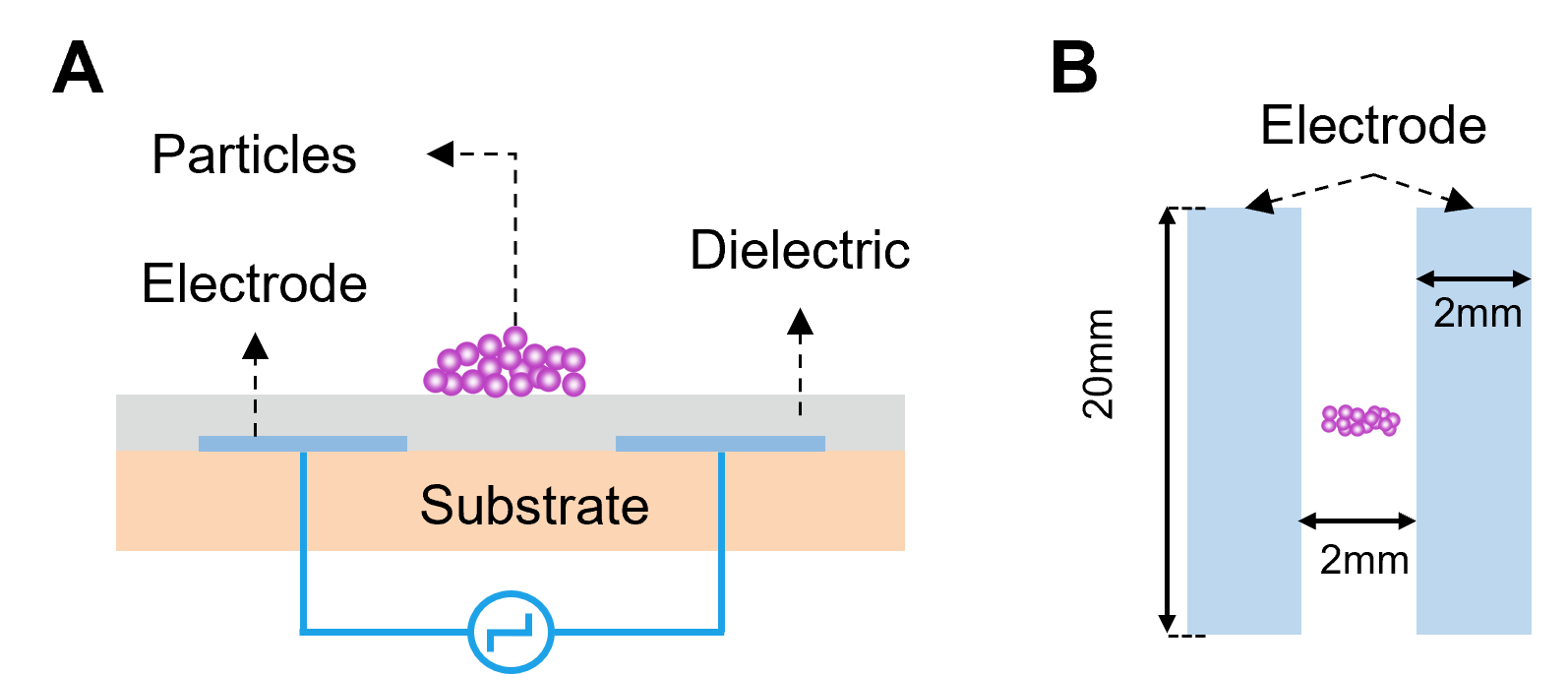
**

**Fig. S1. Initial location of SiO_2_ particles on the glass with two-electrode.** (A) Lateral view. (B) Top view.

**
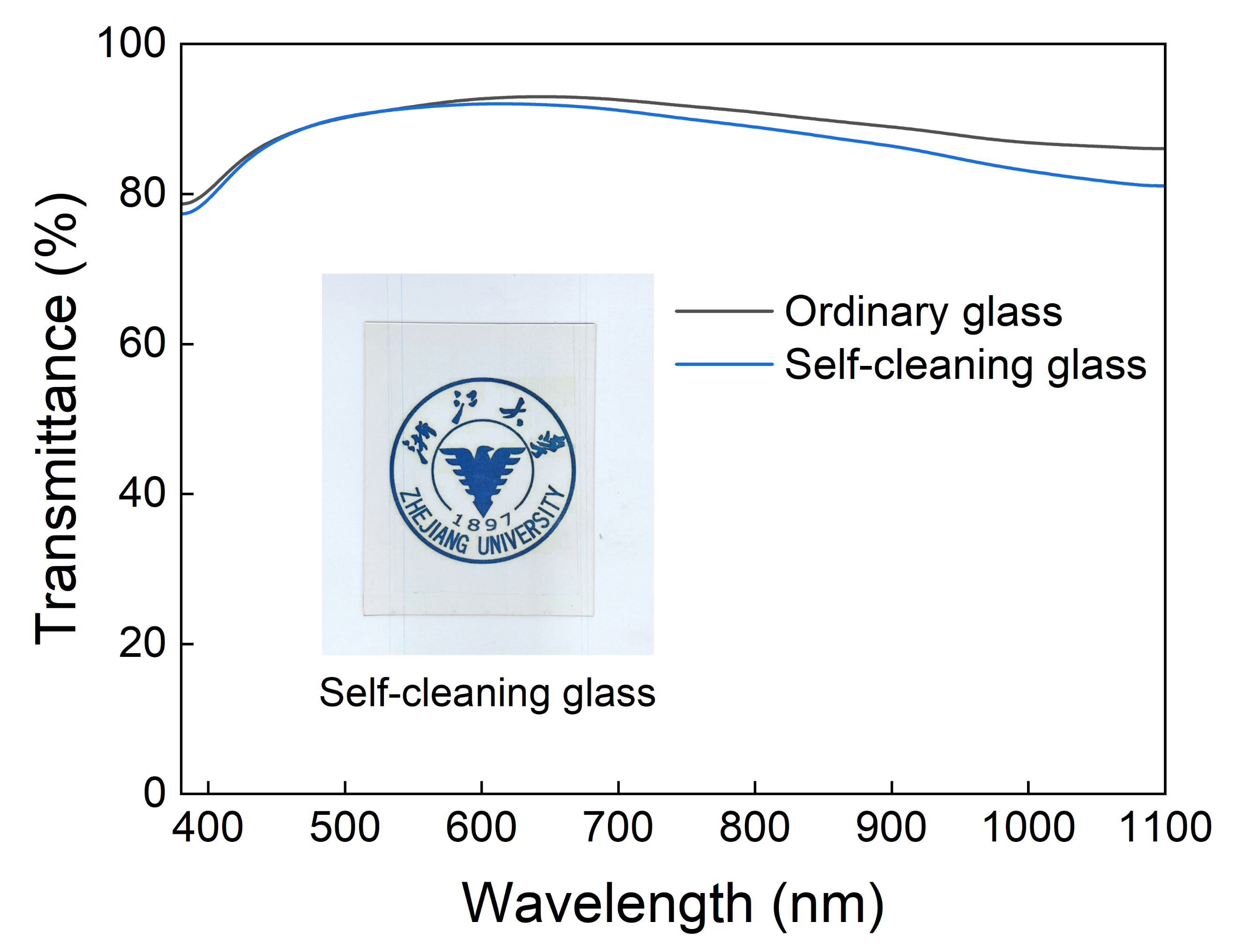
**

**Fig. S2. The light transmittance of the self-cleaning glass and** **ordinary glass.**


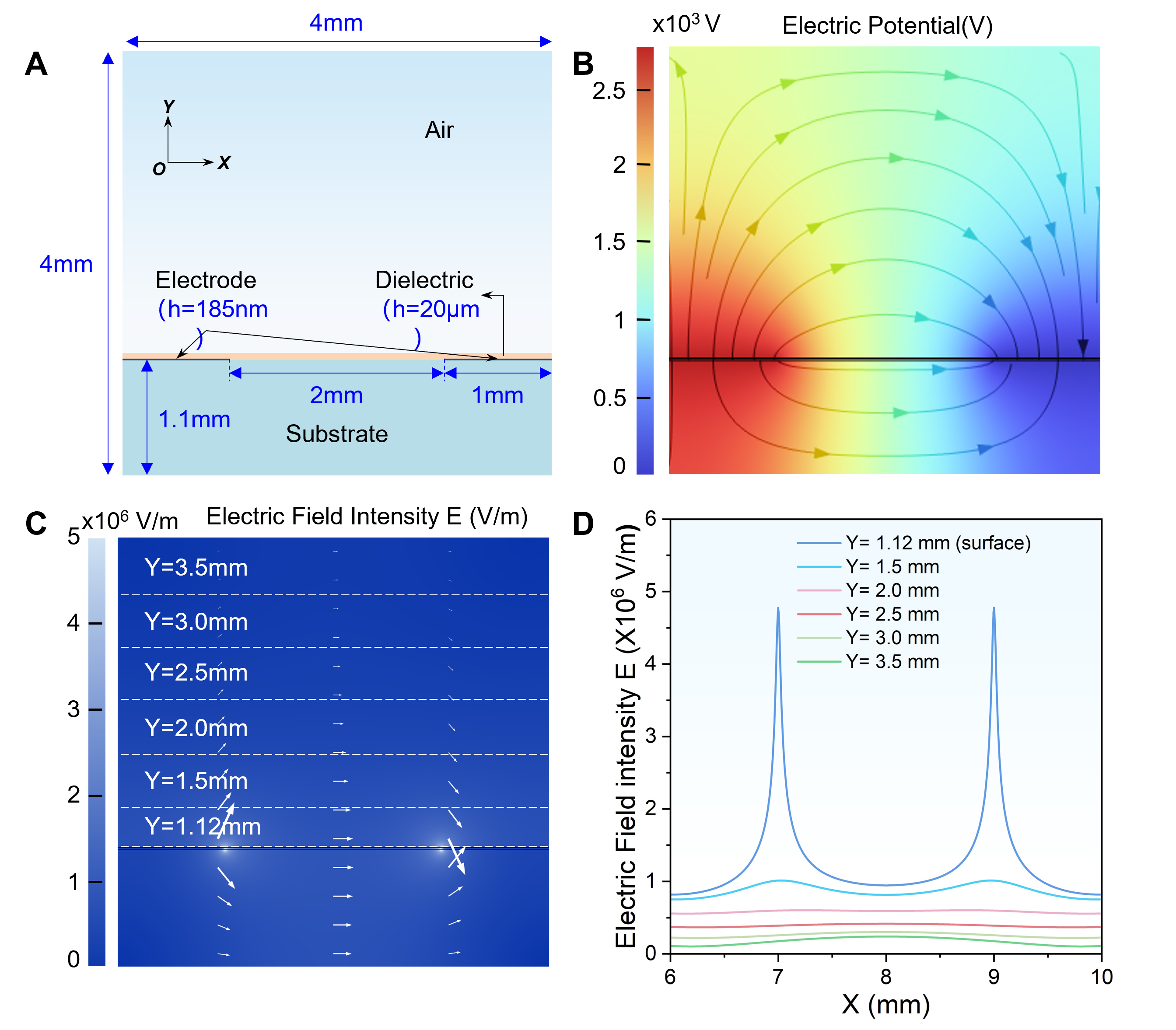


**Fig. S3. Electric field distribution above surface** **of the glass with two-electrode.** (A) Schematic of the simulation model. (B) Electric potential at an applied voltage of 5.5 kV. Please note that the voltage values in this study were all peak-to-peak values (V_p-p_). (C) Overall electric field intensity at an applied voltage of 5.5 kV. (D) Electric field intensity at different heights at an applied voltage of 5.5 kV. The voltages applied to the left and right electrodes were 5.5 kV and 0 V, respectively. The red regions represent areas of high electric potential, consequently causing the charged particles (assuming the charge of particles $q_{p}<0$) to move toward these areas.

Fig. S3(C), (D) shows the distribution of electric field intensity. At the surface, the field strength densities at the edge of the electrodes were significantly higher than that at other places. Specifically, the maximum field strength densities reached 4.78×10^6^ V/m at x=7 mm and 9 mm on the surface, but only 0.95×10^6^ V/m at x=8 mm. This phenomenon arose from the fact that the larger surface curvature of the electrode edge leaded to the higher surface charge density. As the height from the surface gradually increased, the electric field density at the electrode edge and the electrode centres all decreased and the difference between them gradually decreased. Consequently, the particles at the electrode edge will be affected by the stronger Coulomb force and dielectrophoretic force on the surface of the self-cleaning glass.


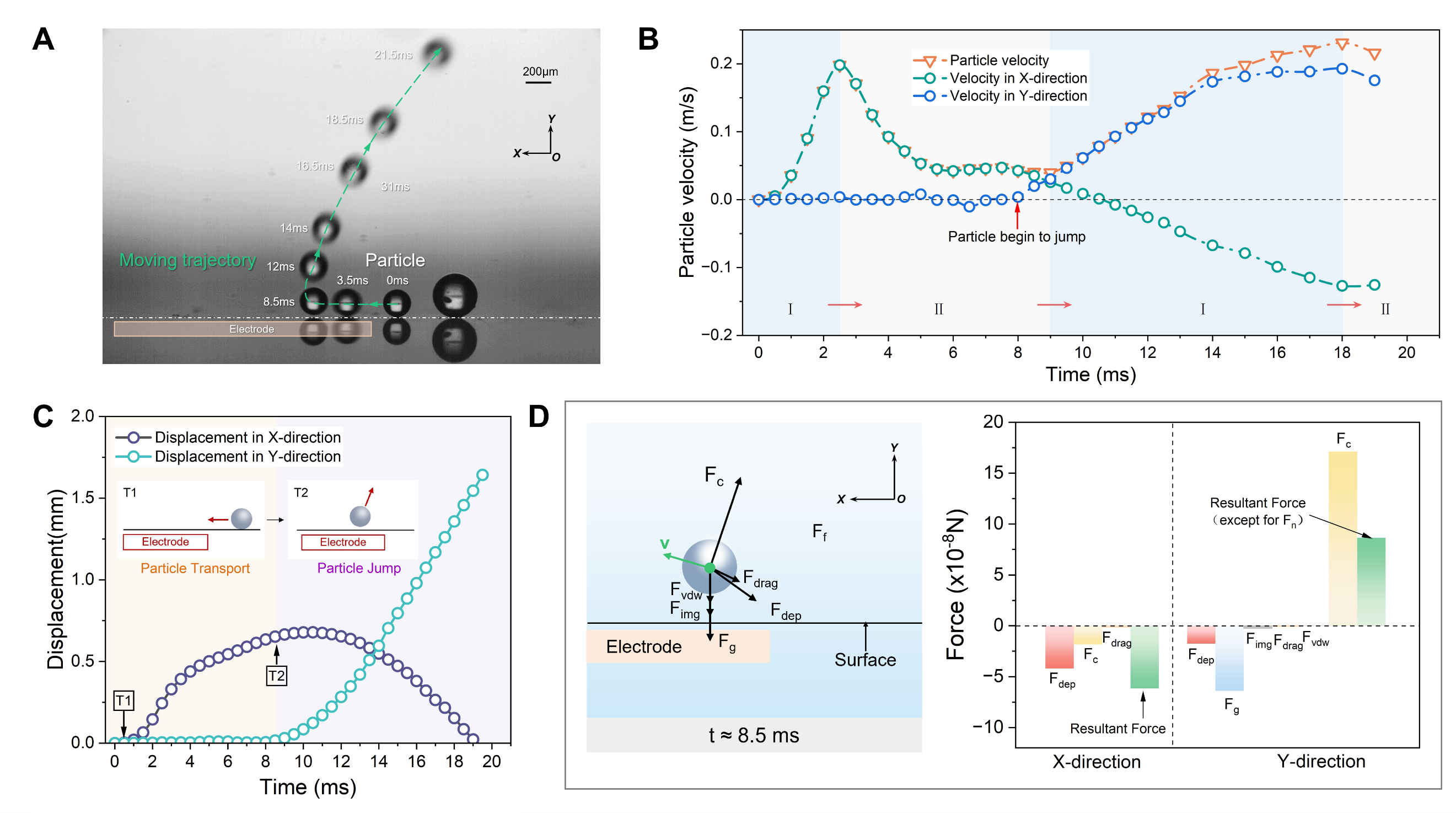


**Fig. S4. A single particle jumped directly in the process of particle transport**. (A) Dynamic behavior of a single particle jumped directly in the process of particle transport (Movie S4). The signal of applied voltage was a single-phase square wave with the voltage of 5.5 kV and frequency of 5 Hz. The diameter of the moving Polymethyl Methacrylate (PMMA) particle was about 221 μm. (B) Particle velocity with the time. Region I represents the acceleration region of particle motion. Region II represents the acceleration region of particle motion. (C) Particle displacement with the time. Light orange background represents that the particles are in the state of particle transport. Light purple background represents that the particles are in the state of particle jump. (D) Force analysis of particle jump at t=8.5 ms.

Fig. S4 shows another typical behavior that a single particle jumped directly in the process of particle transport. Before t=8.5 ms, the particle was in the transport state of particle motion acceleration at t=0-2.5 ms and particle motion acceleration at t=2.5-8.5 ms. The velocities of the particle in the X direction and the Y direction were 0.03519 m/s and 0.01996 m/s at t=8.5 ms, respectively. And the bottom edge of the particle was about 9.608 μm from the surface. It can be seen that the particle at this time was already in jump state due to the change of the electric potential of electrode. As shown in Fig. S4(D), we calculated the forces acting on the particle at t=8.5 ms and found that the total force acting on the particle pointed to the upper right, while the particle velocity was upper left. Therefore, it can be inferred that the particles would experience deceleration to the left in the X direction and then change the direction of motion to the right after t=8.5 ms. But in this process, the particle would continue to accelerate in the Y direction. As illustrated in Fig. S4(B), (C), the above predictions based on the calculation results were consistent with the observed experimental results.


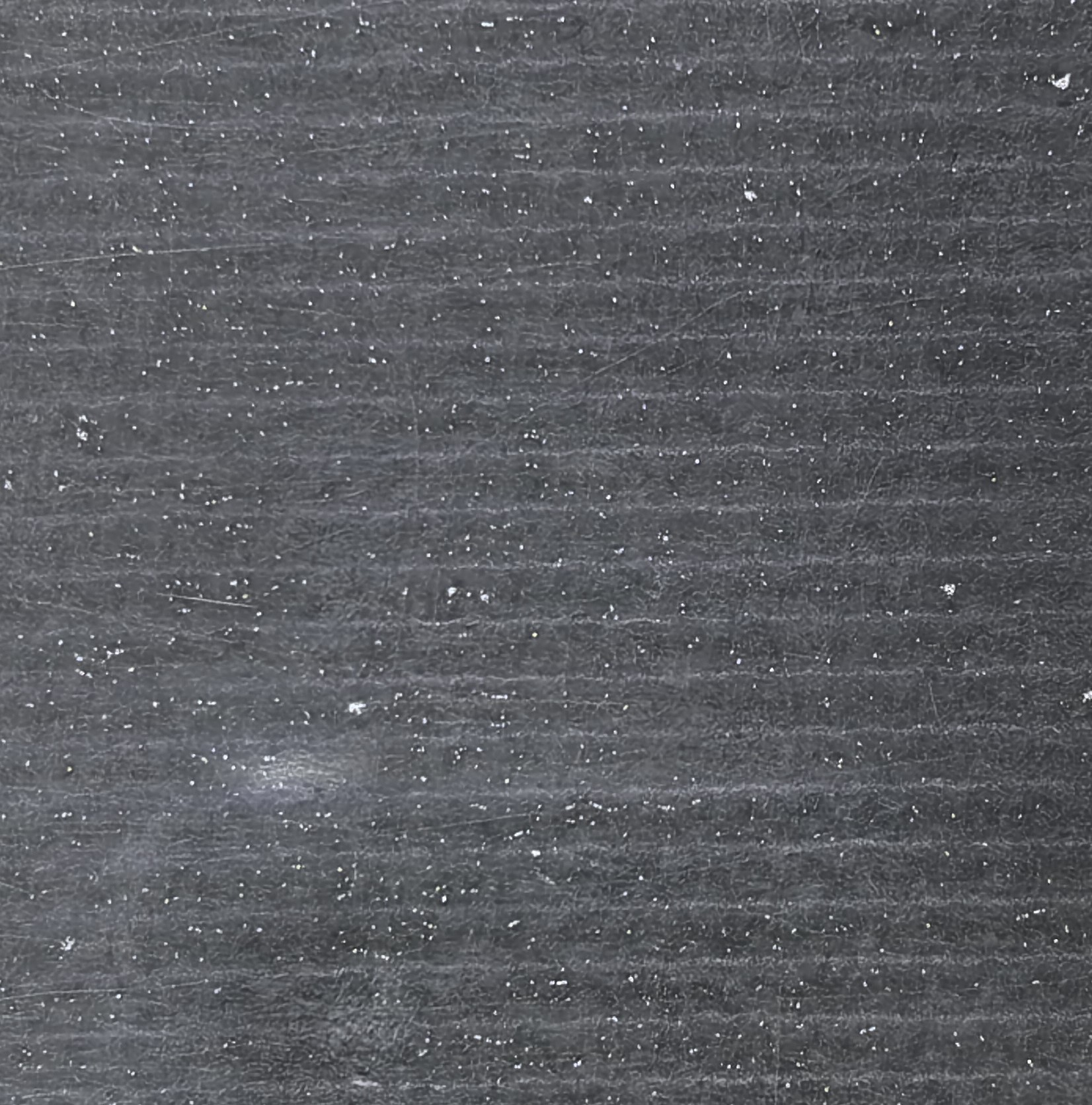


**Fig. S5. Residual particles at the edge of the wavy electrode after self-cleaning process.** The signal of applied voltage was a single-phase square wave with the voltage of 5 kV and frequency of 10 Hz.


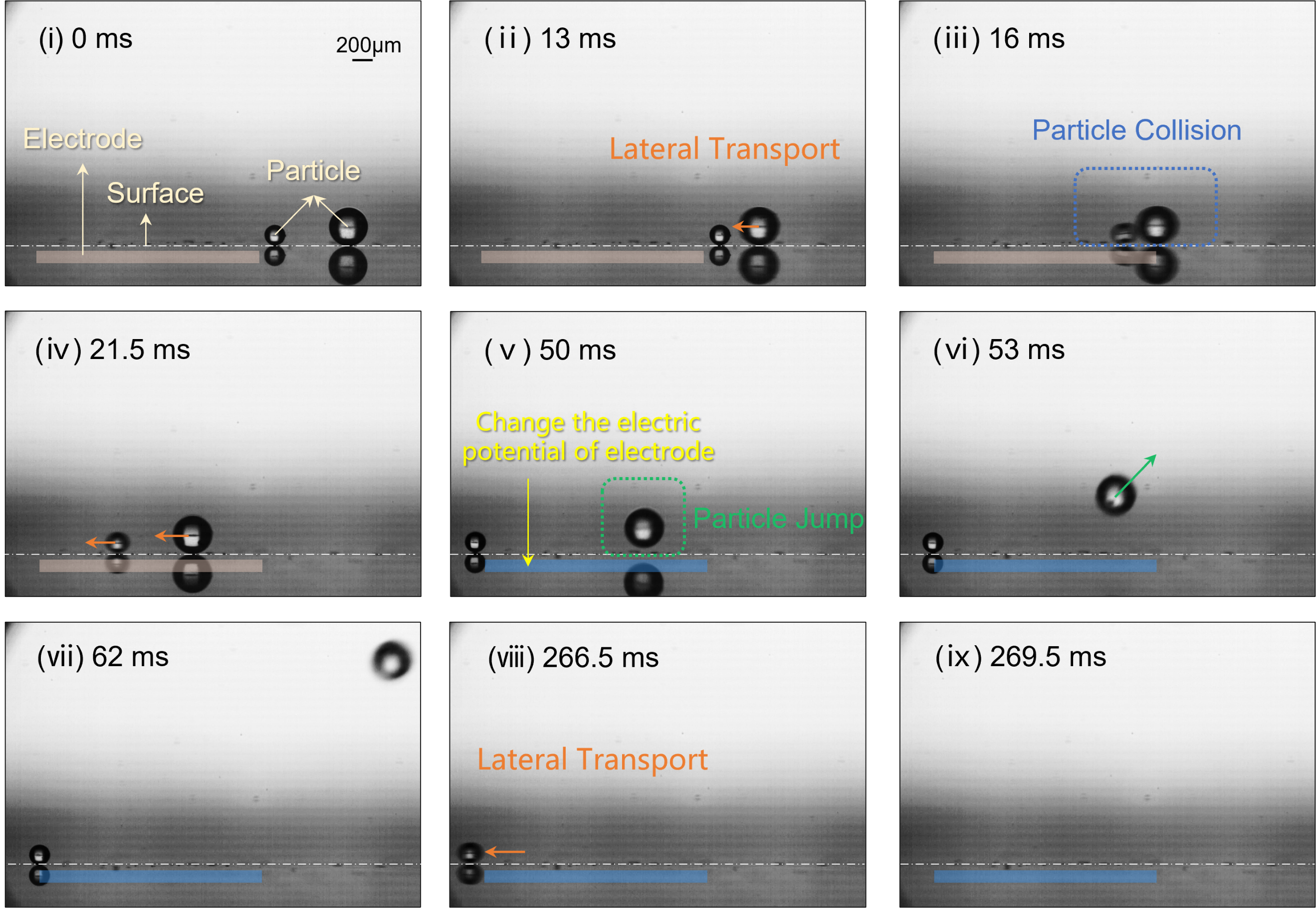


**Fig. S6. Dynamic behavior of collision between residual particles during self-cleaning** **process.** The signal of applied voltage was a single-phase square wave with the voltage of 5.5 kV and frequency of 5 Hz. At t=0 s, the diameter of the left particles was 211 μm, and the diameter of the right particles was 388 μm. The material of the particles was Polymethyl Methacrylate (PMMA).


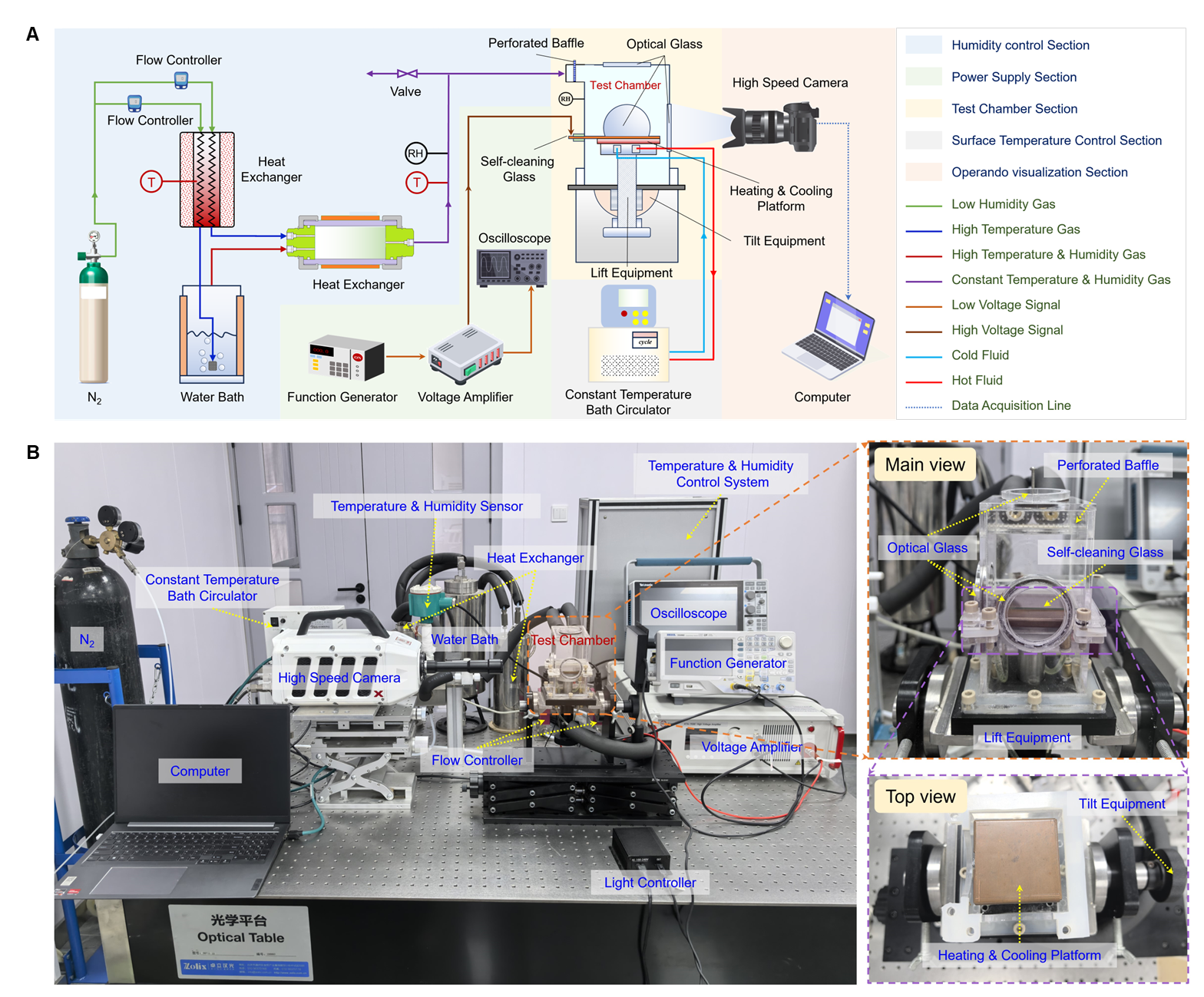


**Fig. S7.** **Schematic of the operando visualization experimental system.** (A) schematic and (B) photograph.

In this study, the experimental system mainly consisted of five main sections: humidity control section, power supply section, test chamber section, surface temperature control section and operando visualization section. To maintain a stable humid environment, we designed a dry and wet gas mixture into the test chamber. The precise control of humidity can be achieved by proportionally controlling the dry and wet volume flow through the flow controller (ACU10FD-LC, ACCU, China). The way of water bath for resulting in humid air was without entraining any water drops[1], which effectively avoided the influence of water droplets on particle motion. The temperature of the gas was constant by electric heating in the whole gas path to prevent water vapor condensation. A temperature and humidity sensor (SM3713M, SONBEST, China) was used to monitor the temperature and humidity of the gas in the gas path in real time. A function generator (DG4062, RIGOL, China) and voltage amplifier (ATA-7030, Aigtek, China) were employed as the power supply. An oscilloscope (3 Series MDO, Tektronix, USA) was used to monitor the waveform and voltage of the signal from the voltage amplifier.

The transparent test chamber was mainly made of organic glass and was equipped with three optical glasses (Hengyang Optical Technology, China) for visualization. There was a heating & cooling platform inside for placing self-cleaning glass and controlling the surface temperature. Constant temperature bath circulator (DC-2006, Tenlin, China) derived cold/hot fluids into the platform to control the surface temperature through heat transfer. In addition, the tilt angle and height of the platform can be adjusted by the tilt equipment and the lifting equipment respectively.

To explore the motion behavior of micron particles in the electric field, we used a high-speed camera (X150, Thousand Eyes Wolf, China) with a telecentric lens (MVL-KT-4-108, Hikvision, China) and micro-distance lens (105 mm f/2.8Sigma, China) for operando observation and recording.

**Supplementary Discussion 1. Details of the main forces affecting particle motion in non-uniform electric field**

**
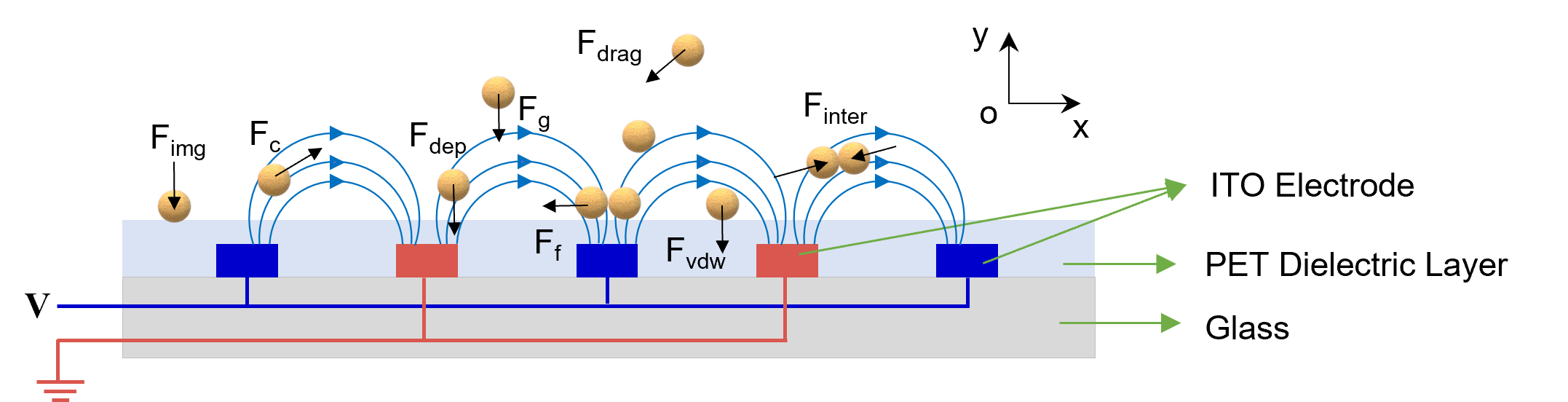
**

**Fig. S8. Force analysis of particles in non-uniform alternating electric field**

Here, we summarized the main forces affecting particle motion in non-uniform electric field: Coulomb force $\vec{F_{c}}$, dielectrophoretic force$\vec{F_{dep}}$, van der Waals force$\vec{F_{vdw}}$, image force $\vec{F_{img}}$, gravitational force$\vec{F_{g}}$, air drag force $\vec{F_{drag}}$ and friction force $\vec{F_{f}}$, as shown in Fig. S8**.**

In an electric field, Coulomb force is one of the main driving forces acting on the charged particles, which can be obtained as:

$$\begin{aligned} \vec{F_{c}}=q_{p}\vec{E}=4\pi r_{p}^{2}\sigma_{s}\vec{E}\#\left( 1 \right) \end{aligned}$$

where $q_{p}$ is the electric charge, $\vec{E}$ is the applied electric field, $r_{p}$ is the particle radius and $\sigma_{s}$ is the surface charge density of the particle. In this study, the value of the surface charge density $\sigma_{s}$ is estimated to be $-1\times{10}^{-6}C/m^{2}$ based on the experiment[2].

Dielectrophoretic force (DEP) results from the interaction between the non-uniform electric field and the dipole moment induced in the dielectric particle[3], which can be expressed as:

$$\begin{aligned} \vec{F_{dep}}=2\pi r_{p}^{3}\varepsilon_{0}\varepsilon_{m}f_{CM}\nabla(E^{2})\#\left( 2 \right) \end{aligned}$$

where $f_{CM}=\left( \varepsilon_{rp}-\varepsilon_{m} \right)/(\varepsilon_{rp}+\varepsilon_{m})$, $\varepsilon_{0}$ is the vacuum permittivity, $\varepsilon_{rp}$ and $\varepsilon_{m}$ are the relative permittivity of the particle and the air medium respectively.

Van der Waals force can be calculated based on a simplified model assuming a smooth spherical particle contacting a nanoscale roughness surface [4,5], which is given by the relation:

$$\begin{aligned} \vec{F_{vdw}}=\frac{-A_{h}r_{p}}{6H_{0}^{2}}\left( \frac{\lambda_{d}^{2}}{\lambda_{d}^{2}+58.14r_{p}RMS}+\frac{H_{0}^{2}}{{(H_{0}+1.817RMS)}^{2}} \right)\#\left( 3 \right) \end{aligned}$$

where $A_{h}$ is the Hamaker constant, $H_{0}$ is the minimum separation distance between the adhering particle and asperity (0.3-0.4 nm), RMS is the root mean square roughness of the surface and $\lambda_{d}$ is the average peak to peak distance between asperities.

The image force was from the image charge induced on the surface of the dielectric layer, which is given by the following relation[6]:

$\begin{aligned} \vec{F_{img}}=-\frac{\varepsilon_{rd}-\varepsilon_{m}}{\varepsilon_{rd}+\varepsilon_{m}}\left( \frac{q_{p}}{16\pi\varepsilon_{0}\varepsilon_{m}} \right)\frac{1}{{(y_{rp})}^{2}}\#\left( 4 \right) \end{aligned}$where $\varepsilon_{rd}$ is the dielectric constant of the dielectric which is a polyester film, $y_{rp}$ is the height of particle above the surface.

Gravitational force can be calculated as:

$$\begin{aligned} \vec{F_{g}}=\frac{4}{3}\pi\rho r_{p}^{3}\vec{g}\#\left( 5 \right) \end{aligned}$$

where $\rho$ is the particle density and g is the gravity acceleration vector.

Air drag force exerted to moving particles can be simplified as[7]:

$$\begin{aligned} \vec{F_{drag}}=-6\pi\eta r_{p}\vec{v_{p}}\#\left( 6 \right) \end{aligned}$$

Where $\eta$ is the dynamic viscosity of air and $v_{p}$ is the particle velocity.

Friction force can be calculated by the normal force and friction coefficient:

$$\begin{aligned} \vec{F_{f}}=\mu\vec{F_{n}}\#\left（ 7 \right） \end{aligned}$$

where $\mu$ is coefficient of static friction and $F_{n}$ is normal force of the surface to the particle.


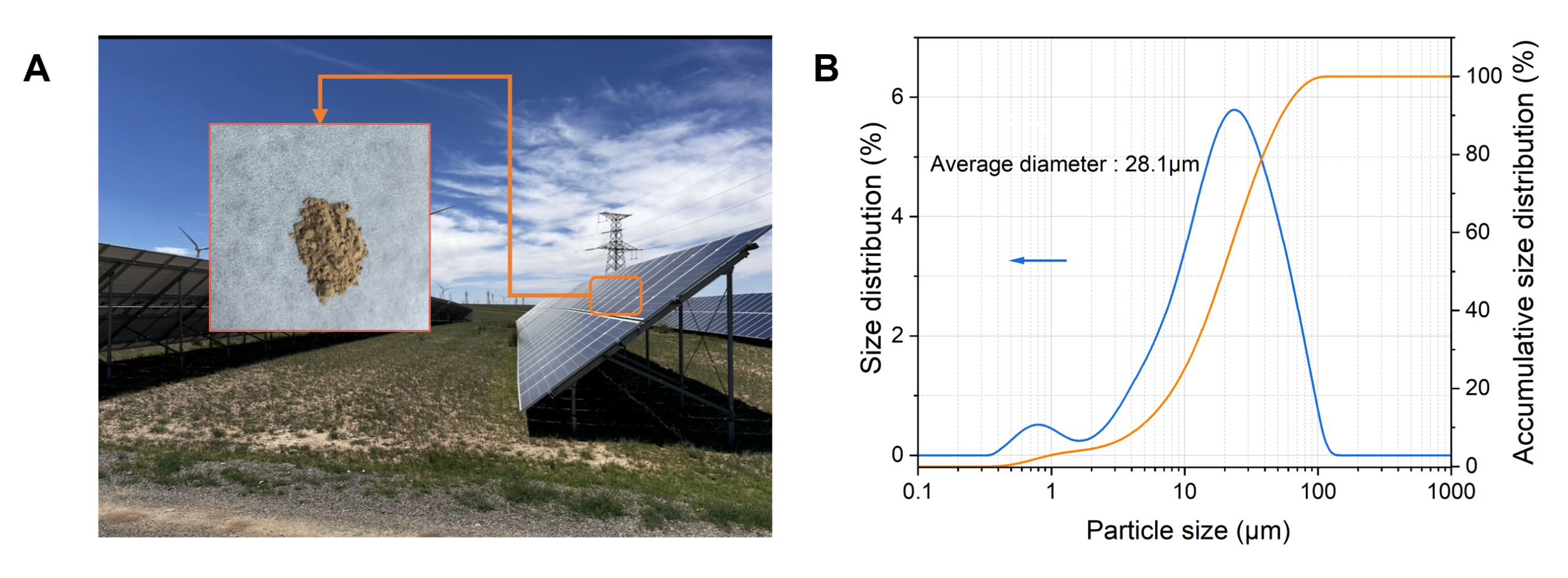


**Fig. S9. Dust from the surface of photovoltaic panels in Nei Mongol Autonomous Region of China**


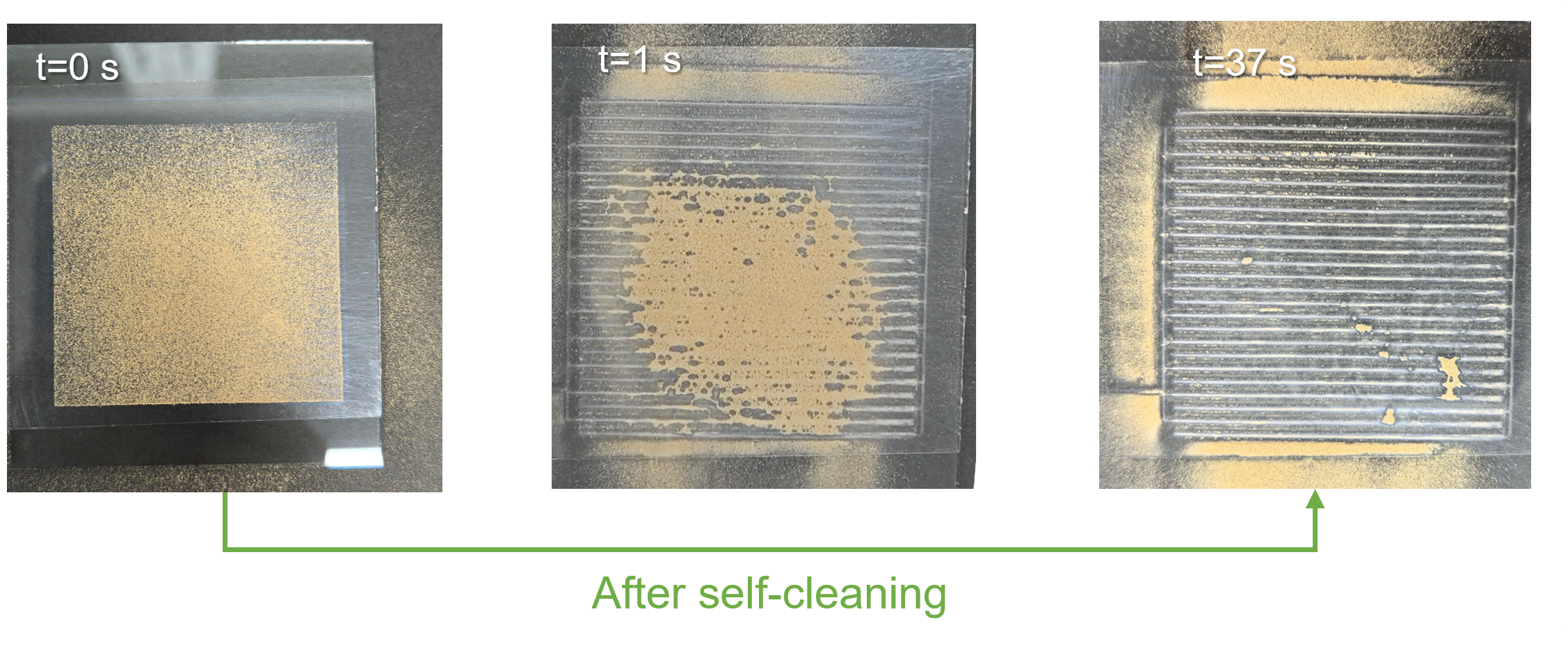


**Fig. S10. Surface self-cleaning demonstration under dust pollution condition.** The signal of applied voltage is a single-phase square wave with the voltage of 5.5 kV and frequency of 10 Hz.

**Supplementary Tables**

**Table S1. The notable advantages of our self-cleaning glass compared with other existing self-cleaning devices from six aspects**. NG means that it is not given.

| **Self-cleaning device driven by electric field** | **Cleaning time (s/cm^2^)** | **Thickness (mm)** | **Cleaning efficiency (%)** | **Particles that can be cleaned** | **Light transmittance (%)** |
| --- | --- | --- | --- | --- | --- |
| Panat et al.[1] | / | / | 99% | Three types (Arizona test dust，Steel particles，Teflon particles) | 90% |
| Baqraf et al.[8] | / | / | 92% | One type  (Dhahran dust) | / |
| Mazumder et al.[9] | 0.496 | / | 93.70% | One type  （JSC-1A dust） | 94.53% |
| Kawamoto et al.[10] | 0.03 | 2 | 99% | One type  (Doha dust) | / |
| This work | 0.4 | 0.62 | 96.92% | Four types (SiO_2_, Al_2_O_3_, PMMA and PVC particles) | 88.64% |

**References**

[1] S. Panat, K.K. Varanasi, Electrostatic dust removal using adsorbed moisture–assisted charge induction for sustainable operation of solar panels, Sci. Adv. 8 (2022) eabm0078. https://doi.org/10.1126/sciadv.abm0078.

[2] A. Zouaghi, N. Zouzou, L. Dascalescu, Assessment of forces acting on fine particles on a traveling-wave electric field conveyor: Application to powder manipulation, Powder Technol. 343 (2019) 375–382. https://doi.org/10.1016/j.powtec.2018.11.065.

[3] A. Zouaghi, N. Zouzou, Numerical modeling of particle motion in traveling wave solar panels cleaning device, J. Electrostat. 110 (2021) 103552. https://doi.org/10.1016/j.elstat.2021.103552.

[4] Capillary forces between surfaces with nanoscale roughness, Adv. Colloid Interface Sci. 96 (2002) 213–230. https://doi.org/10.1016/S0001-8686(01)00082-3.

[5] Y. Yu, J. Cilliers, K. Hadler, S. Starr, Y. Wang, The motion of small particles in electrostatic travelling waves for transport and separation, Powder Technol. 425 (2023) 118587. https://doi.org/10.1016/j.powtec.2023.118587.

[6] T.J. Krinke, K. Deppert, M.H. Magnusson, F. Schmidt, H. Fissan, Microscopic aspects of the deposition of nanoparticles from the gas phase, Aerosol Science (2002). https://doi.org/10.1016/S0021-8502(02)00074-5.

[7] J. Gu, Q. Wang, Y. Wu, L. Feng, G. Zhang, S. Li, L. Tian, W. Yao, Numerical study of particle transport by an alternating travelling-wave electrostatic field, Acta Astronaut. 188 (2021) 505–517. https://doi.org/10.1016/j.actaastro.2021.07.043.

[8] S.A. Baqraf, M.A. Gondal, Mohamed.A. Dastageer, R. Muhammad, A. Al-Aswad, Parametric Optimization of an Unmanned Three-Phase Electrodynamic Dust Shield for Sustainable Photovoltaic Panel Operation for Dusty Environments and Space Applications, ACS Appl. Energy Mater. 5 (2022) 15048–15057. https://doi.org/10.1021/acsaem.2c02712.

[9] M.K. Mazumder, M.N. Horenstein, N.R. Joglekar, A. Sayyah, J.W. Stark, A.A.R. Bernard, S.M. Garner, J.E. Yellowhair, H.Y. Lin, R.S. Eriksen, A.C. Griffin, Y. Gao, R.L. Centra, A.H. Lloyd, Mitigation of Dust Impact on Solar Collectors by Water-Free Cleaning With Transparent Electrodynamic Films: Progress and Challenges, IEEE J. Photovolt. 7 (2017) 1342–1353. https://doi.org/10.1109/JPHOTOV.2017.2721462.

[10] H. Kawamoto, Electrostatic cleaning equipment for dust removal from soiled solar panels, J. Electrostat. 98 (2019) 11–16. https://doi.org/10.1016/j.elstat.2019.02.002.
